# A network approach to investigating the key microbes and stability of gut microbial communities in a mouse neuropathic pain model

**DOI:** 10.1186/s12866-020-01981-7

**Published:** 2020-09-30

**Authors:** Guo-Jie Brandon-Mong, Grace Tzun-Wen Shaw, Wei-Hsin Chen, Chien-Chang Chen, Daryi Wang

**Affiliations:** 1 Biodiversity Research Center, Academia Sinica, 128 Academia Road, Sec. 2, Nankang, Taipei, 11529 Taiwan; 2 Department of Life Science, National Taiwan Normal University, Taipei, Taiwan; 3 Biodiversity Program, Taiwan International Graduate Program, Academia Sinica and National Taiwan Normal University, Taipei, Taiwan; 4 Institute of Biomedical Sciences, Academia Sinica, Taipei, Taiwan; 5 Taiwan International Graduate Program in Molecular Medicine, National Yang-Ming University, Academia Sinica, 128 Academia Road, Sec. 2, Nankang, Taipei, 11529 Taiwan

**Keywords:** Microbiota, Next-generation sequencing, Diversity, Disturbance, Chronic pain, Interaction

## Abstract

**Background:**

Neuropathic pain is an abnormally increased sensitivity to pain, especially from mechanical or thermal stimuli. To date, the current pharmacological treatments for neuropathic pain are still unsatisfactory. The gut microbiota reportedly plays important roles in inducing neuropathic pain, so probiotics have also been used to treat it. However, the underlying questions around the interactions in and stability of the gut microbiota in a spared nerve injury-induced neuropathic pain model and the key microbes (i.e., the microbes that play critical roles) involved have not been answered. We collected 66 fecal samples over 2 weeks (three mice and 11 time points in spared nerve injury-induced neuropathic pain and Sham groups). The 16S rRNA gene was polymerase chain reaction amplified, sequenced on a MiSeq platform, and analyzed using a MOTHUR- UPARSE pipeline.

**Results:**

Here we show that spared nerve injury-induced neuropathic pain alters gut microbial diversity in mice. We successfully constructed reliable microbial interaction networks using the Metagenomic Microbial Interaction Simulator (MetaMIS) and analyzed these networks based on 177,147 simulations. Interestingly, at a higher resolution, our results showed that spared nerve injury-induced neuropathic pain altered both the stability of the microbial community and the key microbes in a gut micro-ecosystem. *Oscillospira*, which was classified as a low-abundance and core microbe, was identified as the key microbe in the Sham group, whereas *Staphylococcus*, classified as a rare and non-core microbe, was identified as the key microbe in the spared nerve injury-induced neuropathic pain group.

**Conclusions:**

In summary, our results provide novel experimental evidence that spared nerve injury-induced neuropathic pain reshapes gut microbial diversity, and alters the stability and key microbes in the gut.

## Background

Neuropathic pain is an abnormal perception of pain [20] that affects 7–10% percent of the human population worldwide [19]. Neuropathic pain can cause increased pain sensitivity to mechanical or thermal stimuli [20]. However, current pharmacological treatments for neuropathic pain are still unsatisfactory because the causes of chronic pain cannot always be established [9]. One of the current treatments is probiotics (live beneficial microbes), and this is still a growing field. For example, the De Simone Formulation probiotic (i.e., *Bifidobacterium breve*, *B. longum*, *B. infantis*, *Lactobacillus plantarum*, *L. paracasei*, *L. delbrueckii* subsp. *Bulgaricus*, *L. acidophilus*, *Streptococcus thermophilus*) alleviated chemotherapy-induced neuropathic pain in an in vitro model, but has not been tested yet in vivo [12]. A recent study attempted to use probiotics (i.e., *Lactobacillus reuteri* lr06 or *Bifidobacterium* bl5b) to alleviate SNI (spared-nerve injury; a traumatic peripheral nerve injury)-induced neuropathic pain, but failed [42].

Host-associated microorganisms have been shown to play important roles in influencing host phenotypes [21]. For example, gut microbiota can regulate visceral pain sensation in mice [58]. An imbalance of beneficial and harmful host-associated microbes (termed dysbiosis [59];) is associated with Alzheimer’s disease [81], and could influence host behaviors such as depression and anxiety [18], and also physiological response such as visceral (internal organ) pain [39]. A recent study showed that the gut microbiota of a host with neuropathic pain after traumatic peripheral nerve injury (spared-nerve injury; SNI) could cause anhedonia (depression-like behavior) to another host through fecal transplantation [84]. However, the underlying questions around topics such as the interactions and stability of the gut microbiota in a traumatic peripheral nerve injury-induced neuropathic pain model and the key microbes involved (i.e., the microbes that play critical roles) have not been answered.

There are generally two types of network inference methods: similarity-based networks and regression-based networks. Similarity-based networks (or co-occurrence networks) that depend on correlation-based methods are often used to infer ecological interactions, but they are rarely efficient for analyzing these interactions [41]. This is because correlation methods can only infer ecological associations [41], and cannot interpret more detailed ecological interactions such as the influence of multiple microbial members on a single member, and vice versa [29]. Regression-based networks depend on generalized Lotka–Volterra (gLV) equations to model temporal changes in more than two taxa [29, 48]. The gLV model is one of the most popular models for microbial community ecology studies [34]. Microbial interaction networks can be constructed using gLV equations to study the influence of microbial members through positive or negative interactions and determine the stability of the microbial communities. For example, Coyte et al. [23] constructed microbial interaction networks using gLV equations and suggested that a higher proportion of negative interactions between microbes indicates a stable microbial community maintained by competitive interactions between microbes. In contrast, a higher proportion of positive interactions between microbes indicates an unstable microbial community maintained by cooperative interactions.

Currently, most disease-associated microbiota studies [13, 28, 33, 57, 85] have inferred unstable microbial communities or dysbiosis from microbial diversity. This is because microbial diversity may have an inverse association with chronic disease and metabolic dysfunction [22, 33]. Consequently, high diversity is generally used to indicate a healthy gut ecosystem [5, 28, 54]. However, some scientists argued that there are problems with using diversity to assess health [11, 45, 70]. Shade [70], for example, proposed that high diversity does not necessarily indicate a stable or healthy microbial community. For example, increased microbial diversity in the vagina may have detrimental consequences such as bacterial vaginosis and preterm birth [11, 30]. In addition, diversity is only an index for the number of taxa in a community and has little ecological value, and a microbial community with high diversity does not necessarily mean that it will remain stable if perturbed [70]. Hence, comparing healthy and diseased states using diversity often leads to contradictory results [45]. Johnson and Burnet [45] call for further studies to investigate these using techniques beyond conventional diversity indices and tackle the issue of a lack of analytical methods to assess microbial composition and functioning.

Our objectives were to determine the interactions in and stability of the gut microbiota in a spared nerve injury-induced neuropathic pain model and the key microbes involved**.** The present study used conventional methods (i.e., alpha & beta diversity and differential abundance), but we could not determine the stability of microbial communities or identify the key microbes in mice with SNI or Sham. On the other hand, we successfully constructed reliable microbial interaction networks using Metagenomic Microbial Interaction Simulator (MetaMIS [72];) based on gLV equations and analyzed the networks. Interestingly, at a higher resolution, our results showed that neuropathic pain after traumatic peripheral nerve injury alters both the stability of microbial community and the key microbes in a gut micro-ecosystem.

## Results

### Pain withdrawal threshold decreased after spared nerve injury (SNI)-induced neuropathic pain

**Fig. 1:**
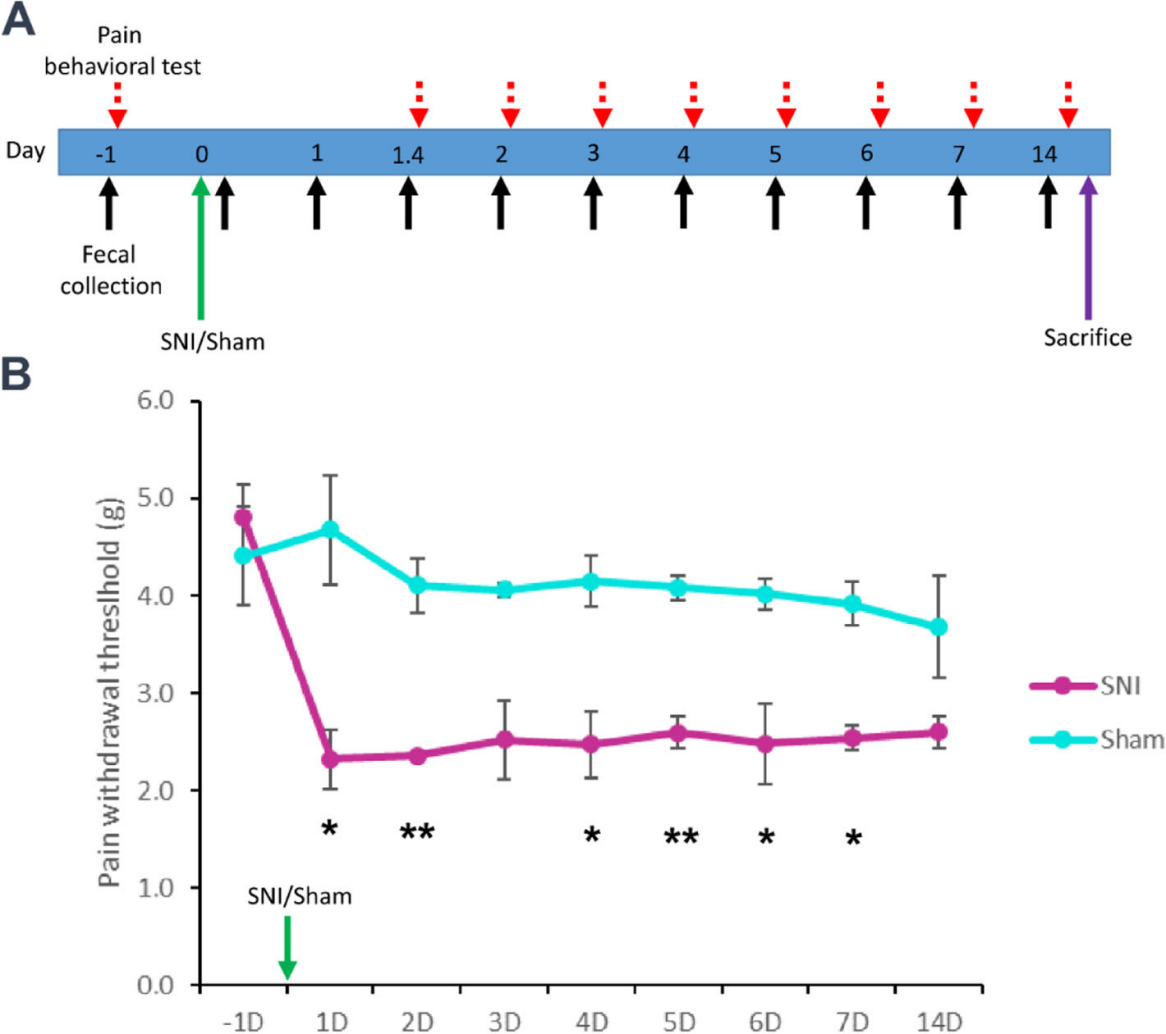
The timeline for fecal collection and pain behavioral test. **a** Day − 1 is the baseline. The SNI and Sham groups were tested on day 0. **b** Student’s T test (* *p* < 0.05, **p < 0.01, SNI group VS Sham group; sample size (n) = 6). Data are shown as mean ± S.E.M

We used the SNI model to investigate the influence of traumatic peripheral nerve injury on the interactions and stability of the gut microbiota and its key microbes. To determine the success of surgeries (i.e., SNI and Sham), we evaluated the pain behavior of mice before and after SNI. The behavioral test was conducted 1 day before surgery (− 1) as a baseline, and 1, 2, 3, 4, 5, 6, 7, and 14 days after surgery. The timeline of the behavioral test and fecal collection is summarized in Fig. 1a. After the traumatic peripheral nerve injury, the paw withdrawal thresholds were significantly lower in the SNI group than in the Sham group on days 1 (*p* value: 0.033), 2 (p value: 0.003), 4 (p value: 0.020), 5 (p value: 0.003), 6 (p value: 0.049), and 7 (p value: 0.012) (Fig. 1b).

### Overview of next-generation sequencing metadata

**Fig. 2:**
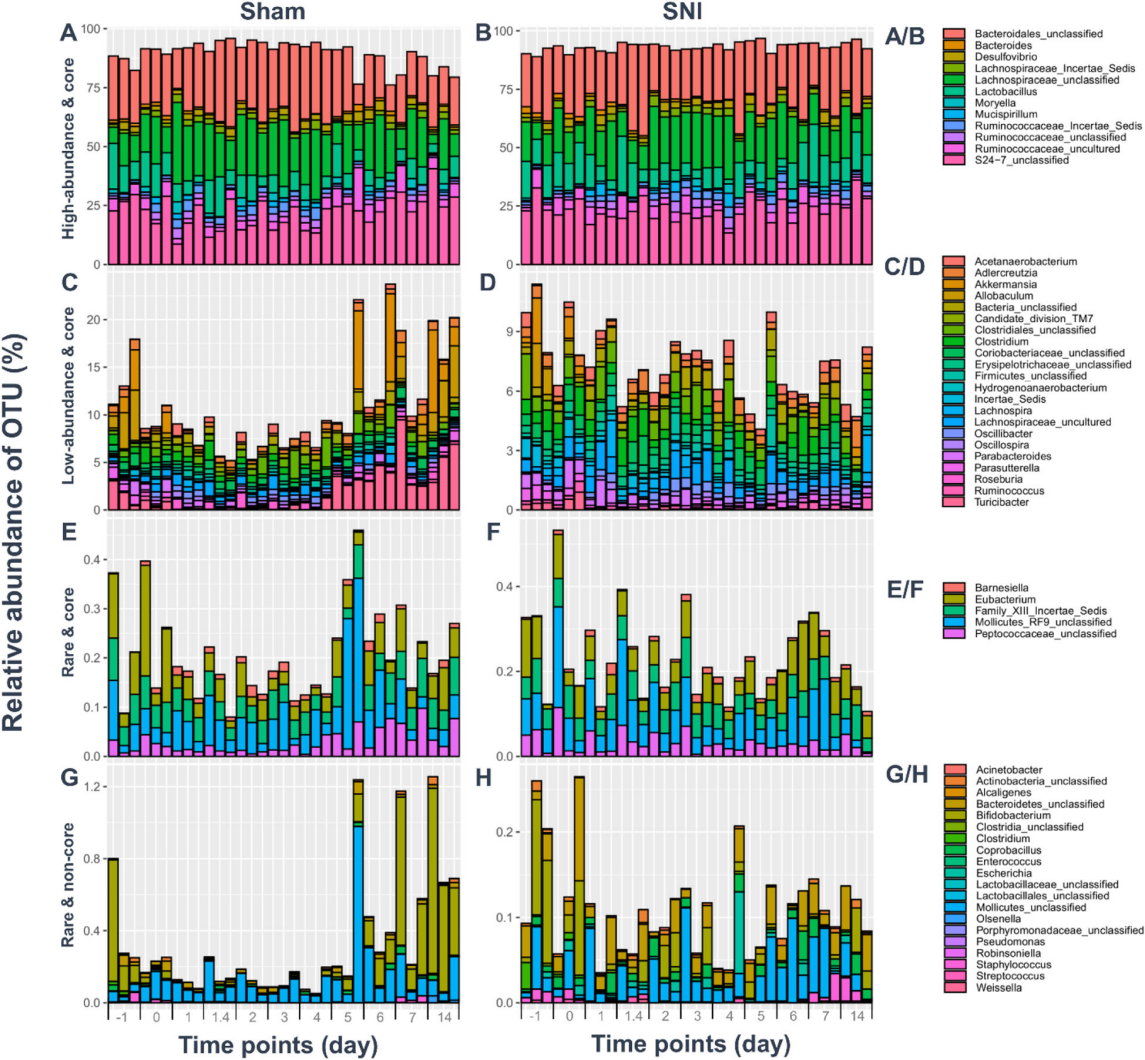
The classification and temporal change of microbial members at the genus level. Microbial members were partitioned based on: (A/B) high-abundance & core; (C/D) low-abundance & core; (E/F) rare & core; (G/H) rare & non-core

To generate time series data and determine the temporal changes in the gut microbiota, we sequenced 66 purified fecal DNA samples, which were collected over 11 time points (1 day before surgery (− 1), and 1, 1.4 (1 day 4 h), 2, 3, 4, 5, 6, 7, and 14 days after surgery; Fig. 1a). In total, we obtained 14,358,275 reads, with an average of 217,550 + 35,517 reads per sample, and around 16% differences in library size. After we filtered and processed the raw data, we determined the number of OTUs (at the genus level; *n* = 59) from each sample, and illustrated them as rarefaction curves (Supplemental Figure 1). All the rarefaction curves of each time-series sample from the SNI and Sham groups reached an asymptote (Supplemental Figure 1). For the differential relative abundance (based on DESeq2; Benjamini-Hochberg adjusted *p*-value), 2 genera were found significantly different between groups on days − 1-0 (*Roseburia*, *p* value: 0.001; *Candidate_division_TM7*, p value: 0.01). Four genera (*Roseburia*, p value: 0.000; *Erysipelotrichaceae_Incertae_Sedis*, *p* value: 0.009; *Mollicutes*_unclassified, *p* value: 0.012; *Peptococcaceae_unclassified*, *p* value: 0.013) were found significantly different between groups on days 1–2. Three genera were found significantly different between groups on days 3–5 (*Turicibacter*, *p* value: 0.000; *Roseburia*, *p* value: 0.004; *Mollicutes_unclassified*, *p* value: 0.005). Eight genera were found significantly different between groups on days 6–14 (*Turicibacter*, *p* value: 0.000; *Allobaculum*, p value: 0.000; *Bifidobacterium*, p value: 0.000; *Akkermansia*, p value: 0.000; *Roseburia*, p value: 0.000; *Mollicutes_unclassified*, p value: 0.009; *Lactobacillus*, p value: 0.013; *Actinobacteria_unclassified*, p value: 0.033). The differential relative abundance of microbes between the SNI and Sham groups is summarized in Supplemental Table 1. To classify the microbial members and track their temporal changes at the genus level, 12 genera were classified as high-abundance and core microbial members: *Bacteroidales_unclassified*, *Bacteroides*, *Desulfovibrio*, *Lachnospiraceae_Incertae_Sedis*, *Lachnospiraceae_unclassified*, *Lactobacillus*, *Moryella*, *Mucispirillum*, *Ruminococcaceae_Insertae_Sedis*, *Ruminococcaceae_unclassified*, *Ruminococcaceae_uncultured*, and *S24–7_unclassified* (Fig. 2a/b); 22 genera were classified as low-abundance and core microbial members: *Acetanaerobacterium*, *Adlercreutzia*, *Akkermansia*, *Allobaculum*, *Bacteria_unclassified*, *Candidate_division_TM7*, *Clostridiales_unclassified*, *Clostridium*, *Coriobacteriaceae_unclassified*, *Erysipelotrichaceae_unclassified*, *Firmicutes_unclassified*, *Hydrogenoanaerobacterium*, *Incertae_Sedis*, *Lachnospira*, *Lachnospiraceae_uncultured*, *Oscillibacter*, *Oscillospira*, *Parabacteroides*, *Parasutterella*, *Roseburia*, *Ruminococcus*, and *Turicibacter* (Fig. 2c/d); 5 genera were classified as rare and core microbial members: *Barnesiella*, *Eubacterium*, *Family_XIII_Incertae_Sedis*, *Mollicutes_RF9_unclassified*, and *Peptococcaceae_unclassified* (Fig. 2e/f); and 20 genera were classified as rare and non-core microbial members: *Acinetobacter*, *Actonobacteria_unclassified*, *Alcaligenes*, *Bacteroidetes_unclassified*, *Bifidobacterium*, *Clostridia_unclassified*, *Clostridium*, *Coprobacillus*, *Enterococcus*, *Escherichia*, *Lactobacillales_unclassified*, *Mollicutes_unclassified*, *Olsenella*, *Porphyromonadaceae_unclassified*, *Pseudomonas*, *Robinsoniella*, *Staphylococcus*, *Streptococcus*, and *Weissella* (Fig. 2g/h).

### Altered gut microbiota diversity and composition

**Fig. 3:**
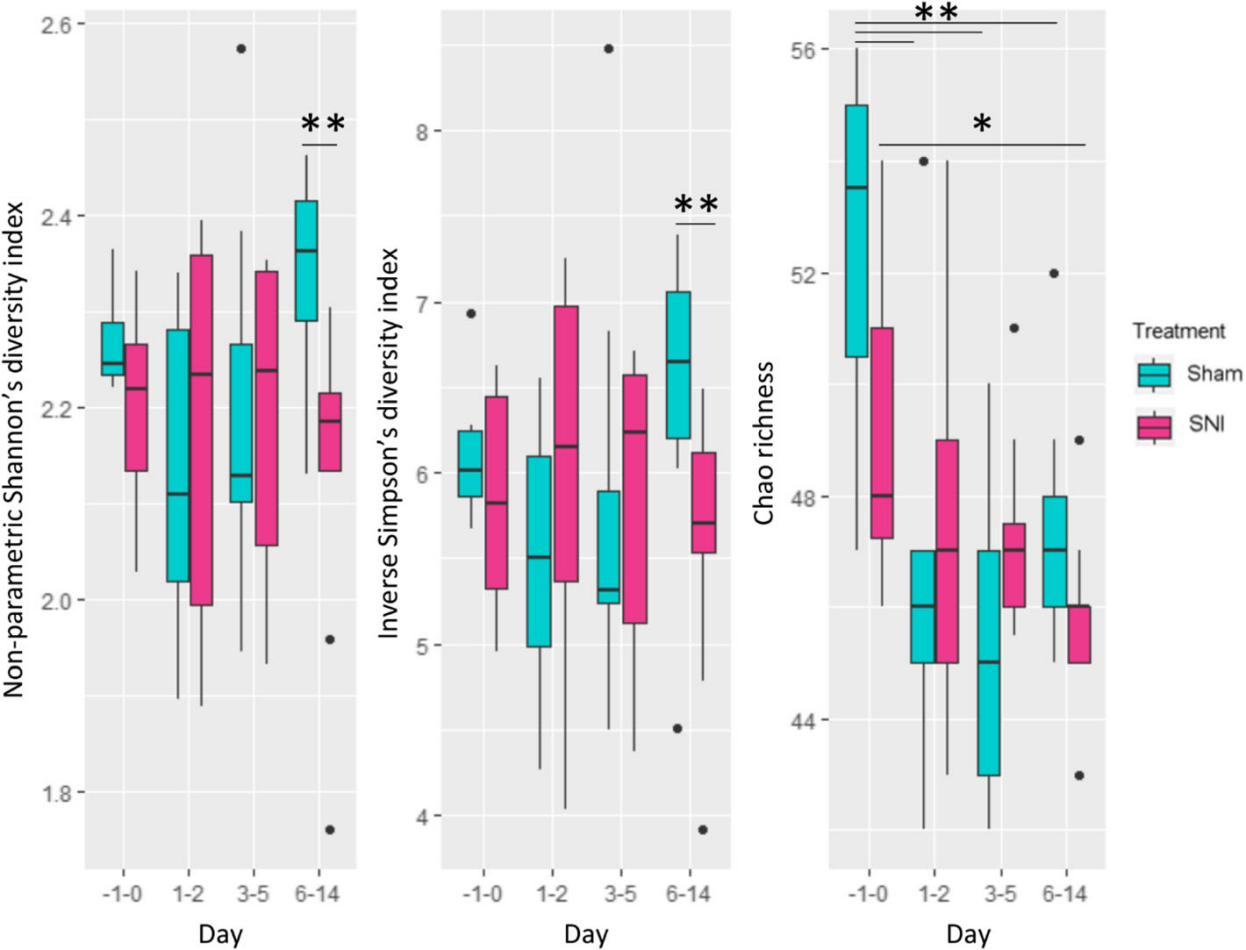
Boxplots of alpha diversity based on non-parametric Shannon’s diversity index, inverse Simpson’s diversity index, and Chao richness. Permutation two sample t-test (* p < 0.05, ** *p* < 0.01). Days − 1–0: *n* = 6; Days 1–2: *n* = 6; Days 3–5: *n* = 9; Days 6–14: *n* = 9

**Fig. 4:**
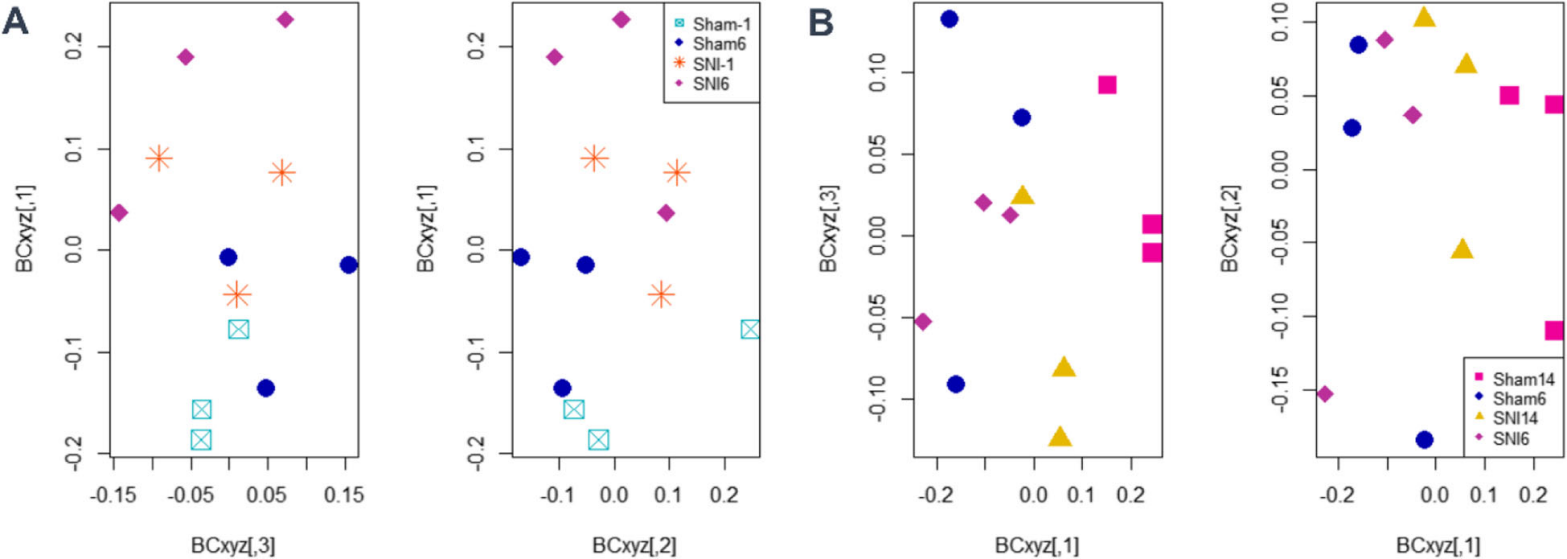
2D plots of 3D NMDS data based on Bray-Curtis dissimilarity. **a** SNI and Sham on day − 1 are not significantly different (PERMANOVA: *p* value = 0.7), thus combined as a group and showed significantly different with SNI and Sham groups on day 6 (PERMANOVA: *p* value = 0.047; *n* = 12); **b** All SNI and Sham groups on days 6 and 14 are significantly different (PERMANOVA: *p* value = 0.048; n = 12)

For phylogenetic indices, alpha diversity (at genus level) was evaluated based on non-parametric Shannon’s diversity index, inverse Simpson’s diversity index, and Chao richness (Fig. 3). For non-parametric Shannon’s diversity index, the genus diversity of the SNI group on days 6–14 was significantly lower (*p* value: 0.004) than that of the Sham group on days 6–14. For the inverse Simpson’s diversity index, the genus diversity of the SNI group on days 6–14 was significantly lower (*p* value: 0.040) than that of the Sham group on days 6–14. For both non-parametric Shannon’s and inverse Simpson’s diversity indexes, the genus diversity of the SNI group on days − 1–0 and days 6–14 was not significantly different. For Chao richness, the genus richnesses of the Sham group on days 1–2, 3–5, and 6–14 were significantly lower (*p* value: 0.006, 0.004, and 0.010, respectively) than those of the Sham group on days − 1 to 0. Likewise, the genus richness of the SNI group on days 6–14 was significantly lower (*p* value: 0.024) than that of the SNI group on days − 1 to 0. For beta-diversity (NMDS based on Bray-Curtis dissimilarity), the composition of SNI on day − 1 was not significantly different compared to the composition of Sham on day − 1, so SNI and Sham on day − 1 was grouped together. The composition of SNI and Sham on day − 1 was significantly different compared to the composition of SNI on day 6 and the composition of Sham on day 6 (*p* value: 0.047; Fig. 4). The composition of SNI and Sham on days 6 and 14 were all significantly different to each other (p value: 0.048; Fig. 4). Interestingly, the composition of SNI on day − 1 was not significantly different compared to the composition of SNI on day 14.

### Stability of microbial interaction networks

**Fig. 5:**
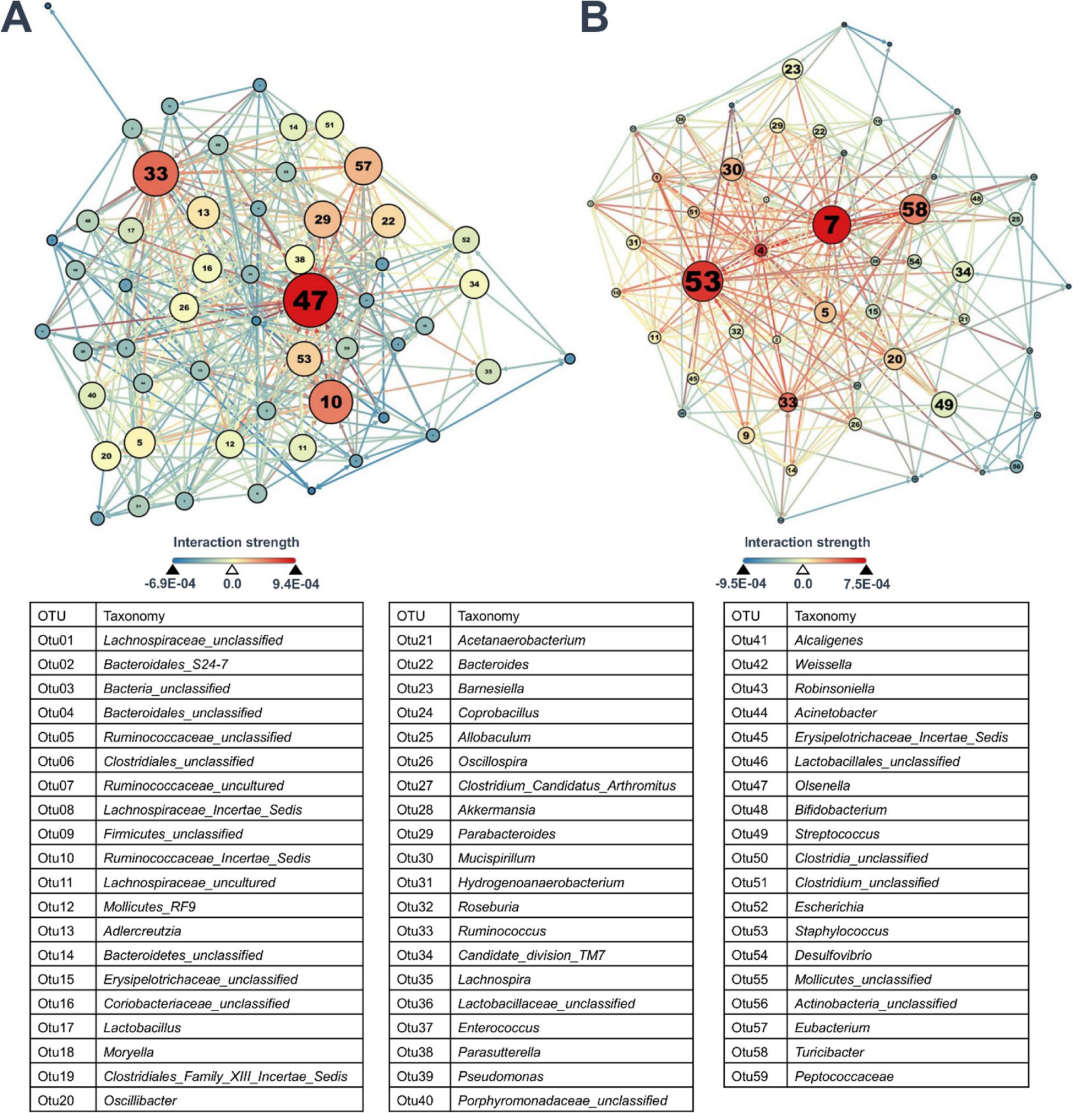
Complex microbial interaction networks based on 177,147 simulations. Each node represents a genus. Node color represents degree, node size represents betweenness centrality, and text size represents closeness centrality. The color of the edge represents the strength of the interaction between two nodes. **a** Microbial interaction network of Sham group; *n* = 3. **b** Microbial interaction network of SNI group; n = 3

To determine the stability of the microbial communities in the Sham and SNI groups, we constructed complex microbial interaction networks and analyzed the proportion of negative and positive interactions in both groups. In the Sham group, the highest proportion of positive interactions was 9.4E-04 (0.0094) and the lowest proportion of negative interactions was − 6.9E-04 (− 0.0069; Fig. 5). In the Sham group, the highest proportion of positive interactions was 7.5E-04 (0.0075), and the lowest proportion of negative interactions was − 9.5E-04 (− 0.0095; Fig. 5). The proportion of positive interactions in the SNI group was significantly higher than that of the Sham group (*p* value = 0.008). The proportion of negative interactions in the SNI group was significantly lower than that of the Sham group (p value = 0.044). Also, the bacterial community in the SNI and Sham groups were highly interacting as a whole (no fragmentation), rather than highly interacting in subpopulations (with fragmentation). The complex microbial interaction networks are summarized in Fig. 5.

### Identification of key microbes

According to the rank that we assigned each genus based on the total rank of betweenness centrality, closeness centrality, and degree centrality, we detected *Oscillospira* and Erysipelotrichaceae_unclassified (rank 1) as the key microbes in the Sham group, followed by *Adlercreutzia* (rank 2) and *Turicibacter* (rank 3) (Supplemental Table 2.1). Oscillospira was identified as the key microbe in the Sham group based on relatively high centrality score (degree: second highest; closeness: fourth highest; betweenness: second highest), with a final rank of 1 (Supplemental Table 2.1). For the SNI group, we detected *Staphylococcus* (rank 1) as the key microbe, followed by *Ruminococcaceae_uncultured* (rank 2) and *Turicibacter* (rank 3) (Supplemental Table 2.2). *Staphylococcus* was identified as the key microbe in the SNI group based on relatively high centrality score (degree: second highest; closeness: third highest; betweenness: the highest), with a final rank of 1 (Supplemental Table 2.2). Multiple linear regression showed that the ranks of the betweenness centrality, closeness centrality, and degree centrality were significantly correlated with the final rank of microbes in both the Sham (*p* value < 0.001; R^2^ = 0.9167) and SNI groups (p value < 0.001; R^2^ = 0.8877; Supplemental Figure 2).

### Interactions between key microbes and other microbes

In the Sham group, *Oscillospira* was influenced by 6 genera through positive interactions and influenced 6 genera through positive interactions. *Oscillospira* was influenced by 5 genera through negative interactions and influenced 13 genera through negative interactions (Table 1 and Supplemental Figure 3A).

**Table 1:** Interactions between *Oscillospira* and other microbes in the Sham group

*Oscillospira* was influenced by other microbes through positive (+) interactions	*Oscillospira* was influenced by other microbes through negative (−) interactions	*Oscillospira* influenced other microbes through positive (+) interactions	*Oscillospira* influenced other microbes through negative (−) interactions
*Barnesiella* *Erysipelotrichaceae_unclassified* *Lactobacillus* *Mollicutes_RF9* *Mollicutes_unclassified* *Streptococcus*	*Clostridiales_unclassified* *Firmicutes_unclassified* *Peptococcaceae* *Ruminococcus* *Ruminococcaceae_unclassified*	*Acetanaerobacterium* *Bacteroidales_unclassified* *Clostridium_Candidatus_Arthromitus* *Lachnospiraceae_unclassified* *Porphyromonadaceae_unclassified* *Ruminococcaceae_Incertae_Sedis*	*Actinobacteria_unclassified* *Akkermansia* *Allobaculum* *Bacteroidales_S24–7* *Bifidobacterium* *Clostridium_unclassified* *Olsenella* *Parabacteroides* *Parasutterella* *Peptococcaceae_unclassified* *Ruminococcaceae_uncultured* *Ruminococcus* *Turicibacter*

In the SNI group, *Staphylococcus* was influenced by 7 genera through positive interactions and influenced 13 genera through positive interactions. *Staphylococcus* was influenced by 7 genera through negative interactions and influenced 3 genera through negative interactions (Table 2 and Supplemental Figure 3B).

**Table 2:** Interactions between *Staphylococcus* and other microbes in the SNI group

*Staphylococcus* was influenced by other microbes through positive (+) interactions	*Staphylococcus* was influenced by other microbes through negative (−) interactions	*Staphylococcus* influenced other microbes through positive (+) interactions	*Staphylococcus* influenced other microbes through negative (−) interactions
*Bacteroides* *Clostridium_unclassified* *Erysipelotrichaceae_Incertae_Sedis* *Lachnospira* *Parabacteroides* *Ruminococcus* *Turicibacter*	*Bacteroidales_unclassified* *Candidate_division_TM7* *Eubacterium* *Lachnospiraceae_unclassified* *Mollicutes_unclassified* *Roseburia* *Ruminococcaceae_Incertae_Sedis*	*Bacteria_unclassified* *Clostridiales_unclassified* *Desulfovibrio* *Firmicutes_unclassified* *Hydrogenoanaerobacterium* *Lachnospiraceae_Incertae_Sedis* *Lachnospiraceae_unclassified* *Lachnospiraceae_uncultured* *Mucispirillum* *Oscillibacter* *Oscillospira* *Ruminococcaceae_Incertae_Sedis* *Ruminococcaceae_unclassified*	*Akkermansia* *Bacteroidales_S24–7* *Lactobacillus*

### Interactions between potential probiotics and other microbes

In the Sham group, potential probiotics such as *Lactobacillus* were influenced by 6 genera through positive interactions and influenced 5 genera through positive interactions. *Lactobacillus* was influenced by 1 genus through negative interactions and influenced 6 genera through negative interactions (Table 3 and Supplemental Figure 3C). Overall, there were two potential probiotics (*Lactobacillus* and *Bifidobacterium*) in this network (Table 3 and Supplemental Figure 3C).

**Table 3:** Interactions between *Lactobacillus* and other microbes in the Sham group

*Lactobacillus* was influenced by other microbes through positive (+) interactions	*Lactobacillus* was influenced by other microbes through negative (−) interactions	*Lactobacillus* influenced other microbes through positive (+) interactions	*Lactobacillus* influenced other microbes through negative (−) interactions
*Clostridium_unclassified* *Eubacterium* *Firmicutes_unclassified* *Parabacteroides* *Parasutterella* *Ruminococcus*	*Clostridia_unclassified*	*Desulfovibrio* *Lachnospiraceae_uncultured* *Oscillibacter* *Ruminococcaceae_Incertae_Sedis* *Ruminococcaceae_unclassified*	*Adlercreutzia* *Bifidobacterium* *Coprobacillus* *Porphyromonadaceae_unclassified* *Ruminococcus* *Turicibacter*

In the Sham group, *Bifidobacterium* was influenced by 2 genera through positive interactions and influenced 2 genera through positive interactions. *Bifidobacterium* was influenced by 7 genera through negative interactions and influenced 1 genus through negative interactions (Table 4 and Supplemental Figure 3E). Overall, there were three potential probiotics (*Bifidobacterium*, *Lactobacillus*, and *Enterococcus*) in this network (Table 4 and Supplemental Figure 3E).

**Table 4:** Interactions between *Bifidobacterium* and other microbes in the Sham group

*Bifidobacterium* was influenced by other microbes through positive (+) interactions	*Bifidobacterium* was influenced by other microbes through negative (−) interactions	*Bifidobacterium* influenced other microbes through positive (+) interactions	*Bifidobacterium* influenced other microbes through negative (−) interactions
*Adlercreutzia* *Turicibacter*	*Lactobacillus* *Bacteroidales_S24–7* *Erysipelotrichaceae_unclassified* *Barnesiella* *Enterococcus* *Mollicutes_unclassified* *Oscillospira*	*Acinetobacter* *Ruminococcus*	*Coriobacteriaceae_unclassified*

In the SNI group, *Lactobacillus* was influenced by 3 genera through positive interactions and influenced 5 genera through positive interactions. *Lactobacillus* was influenced by 3 genera through negative interactions and did not influence any other microbes through negative interactions (Table 5 and Supplemental Figure 3D). Overall, there was only one potential probiotic (*Lactobacillus*) in this network (Table 5 and Supplemental Figure 3D).

**Table 5:** Interactions between *Lactobacillus* and other microbes in the SNI group

*Lactobacillus* was influenced by other microbes through positive (+) interactions	*Lactobacillus* was influenced by other microbes through negative (−) interactions	*Lactobacillus* influenced other microbes through positive (+) interactions	*Lactobacillus* influenced other microbes through negative (−) interactions
*Hydrogenoanaerobacterium* *Parasutterella* *Staphylococcus*	*Bacteroidales_unclassified* *Barnesiella* *Lachnospiraceae_unclassified*	*Bacteria_unclassified* *Clostridiales_unclassified* *Desulfovibrio* *Lachnospiraceae_uncultured* *Parabacteroides*	None

In the SNI group, *Bifidobacterium* was influenced by 1 genus through positive interactions and influenced 7 genera through positive interactions. *Bifidobacterium* was influenced by 5 genera through negative interactions and influenced 1 genus through negative interactions (Table 6 and Supplemental Figure 3F). Overall, there was only one potential probiotic (*Bifidobacterium*) in this network (Table 6 and Supplemental Figure 3F).

**Table 6:** Interactions between *Bifidobacterium* and other microbes in the SNI group

*Bifidobacterium* was influenced by other microbes through positive (+) interactions	*Bifidobacterium* was influenced by other microbes through negative (−) interactions	*Bifidobacterium* influenced other microbes through positive (+) interactions	*Bifidobacterium* influenced other microbes through negative (−) interactions
*Acetanaerobacterium*	*Akkermansia* *Clostridium_Candidatus_Arthromitus* *Lachnospiraceae_uncultured* *Ruminococcus* *Turicibacter*	*Acetanaerobacterium* *Barnesiella* *Candidate_division_TM7* *Erysipelotrichaceae_unclassified* *Eubacterium* *Mollicutes_RF9* *Ruminococcaceae_uncultured*	*Allobaculum*

## Discussion

There is currently a discussion in the academic community about whether microbial diversity can be used to determine the health of a gut environment [11, 45, 70]. In our study, we first analyzed microbial diversity to determine the influence of neuropathic pain on the health conditions (i.e., stability) of hosts’ gut environments. Even though 2 genera were found significantly different between the SNI and Sham groups on days − 1-0 (baselines), which might due to stochastic events, the alpha and beta diversity between groups on days − 1-0 were not significantly different. The changes in microbial diversity (i.e., Shannon and Simpson’s diversity indices; alpha diversity) in our study were similar to those in a previous study [84], in which the microbial diversity in the SNI group was significantly lower than the microbial diversity in the Sham group at the end of the experiment. In contrast, another previous study showed that the microbial diversity between the SNI group with a normal level of vitamin D and the Sham group with a normal level of vitamin D was not significantly different [38]. Interestingly, the results of the alpha diversity (i.e., the genus diversity of the SNI group on days − 1-0 and days 6–14 was not significantly different but the genus diversity of SNI group on days 6–14 was significantly lower than the genus diversity of Sham group on days 6–14) and beta-diversity (i.e., the composition of SNI on day − 1 was not significantly different compared to the composition of SNI on day 14) in our study made determining the stability of the microbial community complicated for both groups. To overcome these impediments, we constructed and analyzed complex microbial interaction networks. In accordance with Coyte et al. [23], we found that the Sham group may have a stable gut microbial community due to its high proportion of competitive interactions. In contrast, the SNI group may have an unstable gut microbial community due to its high proportion of cooperative interactions. Even though cooperative interactions may enhance metabolic efficacy, a decrease in the abundance of certain microbes may affect other cooperating microbes, eventually destabilizing the entire microbial community [23]. Our results are in accordance with studies [7, 62, 79] that suggest that the interactions in the mammalian microbiota are generally competitive and exploitative. For instance, Venturelli et al. [79] co-cultured a 12-species community and found that 68% of the total interactions were competitive and ammensal, and only 5% were cooperative and commensal. Furthermore, Heinken and Thiele [40] simulated an 11-species community and found that cooperative and commensal interactions were rare (~ 10% of total interactions).

*Oscillospira*, which was classified as a low-abundance and core microbe, was identified as the key microbe (rank 1) in the Sham group, followed by *Adlercreutzia* (rank 2). Previous studies have shown that *Oscillospira* is associated with health [26, 49, 77, 80]. For example, *Oscillospira* is enriched in *Christensenella minuta*-altered gut microbial community, which is involved in the promotion of leanness in mice [35]. In a study conducted on human adult monozygotic twins, *Oscillospira* was significantly more abundant in the non-obese twin (lower BMI [77];); another study, however, found the relative abundance of *Oscillospira* to be significantly lower in obese humans [80]. Gophna et al. [36] suggested that *Oscillospira* is associated with leanness and is probably involved in the promotion of leanness because *Oscillospira* uses host glycans as a food source and causes the host to spend metabolic energy to resynthesize degraded glycoproteins (e.g., gut mucins). Along with its association with leanness, *Oscillospira* can produce butyrate [36], which can protect a host against infections [51] and enhances sleep [75].

Like *Oscillospira*, *Adlercreutzia* is also associated with health. According to Euzéby [27], the genus *Adlercreutzia* contains only one species: *Adlercreutzia equolifaciens*, an equol-producing bacterium [61]. Equol is a secondary metabolite of daidzein with a higher anti-carcinogenic activity than daidzein [53]. Moreover, equol can protect macrophages from oxidative stress caused by microbial lipopolysaccharides, and increase host antioxidants and cytokines [37]. Liu et al. [53] showed that the abundance of *Adlercreutzia* decreases in mice with high fat diets. In our study, these beneficial microbes (i.e., *Oscillospira* and *Adlercreutzia*) were the top two key microbes in the Sham group, and this may further support the idea that the microbial interaction network constructed in this study was reliable and the gut ecological environment in the Sham group was stable and healthy.

Along with the interactions among microbes, studies have also shown that the gut microbiota co-evolved with a host to form a mutually beneficial relationship [2, 3, 17]. Hence, the gut microbiota can interact with the host immune system [32]. For instance, the immune system maintains a stable and healthy gut microbiota by promoting the growth of beneficial gut microbes [52, 67], whereas a stable and healthy gut microbiota can produce molecular signals that contribute to the development of immune cells and support proper immune responses [52, 67] to defend against pathogens [17]. Hence, along with suffering from chronic pain, the mice with SNI in our study might also have suffered from a weakened immune system due to unstable microbiota communities, probably making them more susceptible to pathogen infection.

Numerous studies showed that the supplementation of probiotics is effective for combating various pathogens [4, 24, 65] and reducing anxiety and depression-related behavior in mice [10]. Huang et al. [42] attempted to treat neuropathic pain in rats with oral supplementation of probiotics (i.e., *Lactobacillus reuteri* LR06 or *Bifidobacterium* BL5b), but failed. Along with the reasons provided by Huang et al. [42], we speculate that the treatment failed because key microbes in the rats with neuropathic pain were influenced by and cooperated with the probiotics for survival, and these attenuated the analgesic effects of the probiotics. Interestingly, *Staphylococcus*, the key microbe in the SNI group, influenced *Lactobacillus* through cooperative interactions in our study.

*Staphylococcus*, which was classified as a rare and non-core microbe, was identified as the key microbe (rank 1) in the SNI group. *Staphylococcus* are infamous for their inflammatory effects on hosts; for example, *Staphylococcus aureus* can cause abscesses, cellulitis, osteomyelitis [8, 56], and dermatitis [63]; both *S. aureus* and *S. epidermidis* can cause septic arthritis [44, 47]; and *S. saprophyticus* can cause pyelonephritis [15, 43]. *S. haemolyticus*, *S. intermedius*, *S. lugdunensis*, *S. schleiferi*, and *S. warneri*, however, are infrequent pathogens [31]. Other than inflammatory effects, *S. aureus* can also cause mechanical and thermal hyperalgesia at the hind paw of mice due to pore-forming toxins produced by *S. aureus* and subsequently cause nociceptor neuron activation [8]. Even though *Staphylococcus* was rare in our study, *Staphylococcus* might increase in abundance after 2 weeks because *Staphylococcus* is an opportunist with fast community succession [69]. Rare microbes in certain favorable conditions may occasionally become dominant [71] and more important in providing certain functional traits [16].

Every method is different and has its own limitations. Some studies removed rare microbial taxa from their analyses—e.g., rare OTUs that might be sequencing artifacts were removed before downstream analyses [60], and rare OTUs were removed from network analyses to reduce false positive results [82]. Rarefying data with a big (around 10 times or 1000%) difference in the average sequencing library size can also reduce false positive results [83]. However, rarefying data produces false negative results because part of the data is discarded [83]. To retain the rare microbial taxa in our study, we did not remove rare OTUs nor did we rarefy our data to equalize sequencing depth. In fact, the differences among average sequencing library sizes in our study were very small (around 16%). Chen et al. [16] showed that rare microbes might have a more important role in driving multiple functions than do dominant microbes. For instance, rare microbes may influence and change the composition of gut microbiota after a diet change [6]. Rare microbes have also been found to play important roles among host-associated microbes in both animals and plants and enhance host immune systems [46, 74, 78]. The current understanding of the ecological role of host-associated rare key microbes in an unstable gut microbial community is still in its infancy and should be investigated further.

## Conclusion

In summary, our results provide novel experimental evidence that traumatic peripheral nerve injuries may alter the microbial diversity and stability in the gut, as well as the composition of its key microbes. Nevertheless, the complex microbial interaction networks in our study were simulated based on theoretical analyses. Hence, more biological experiments are required to validate and support our results.

## Methods

### Animal preparation

All animal procedures conformed to the guidelines specified by the National Institutes of Health and the Institutional Animal Care and Utilization Committee (IACUC), Academia Sinica (Taipei, Taiwan), including the guidelines for the Replacement, Refinement & Reduction of Animals in Research. IACUC approved protocol: 18-12-1246. C57 black 6 (C57BL/6) mice were ordered from the National Laboratory Animal Center (Taipei, Taiwan) and quarantined for a week before any experiment. Six female mice 8 to 12 weeks old were used for the experiment (3 for SNI group, 3 for Sham group). We performed the experiments on female mice because female mice are less aggressive to other female mice than male mice [76]. We considered this stress as a confounder in our experiments that might alters the gut microbial diversity and composition. Mice were held under a 12:12-h light-dark cycle with food and water available ad libitum. At the end of the experiment, the mice were anesthetized with isoflurane and a mixture of ketamine and xylazine, and sacrificed using cervical dislocation.

### Spared-nerve injury model

Surgical procedures were performed under isoflurane anesthesia (liquid inhalation; Aerane, Baxter, USA) according to previous studies [14, 73]. After a skin and muscle incision on the left hind limb, the sural nerve and peroneal nerve were tightly ligated using a gamma sterile monofilament with a 3/8 circle taper point (UNIK, Taiwan), then transected. The tibial nerve was not ligated or transected. For the Sham controls, the sural, peroneal, and tibial nerves were exposed but were not ligated or transected. Finally, the skin and muscles were closed using a silk suture.

### Behavioral test

A double-blind randomized behavioral test was conducted at room temperature (approximately 25 °C) and only during the daytime. Before testing behavior, the mice were habituated on an elevated wire mesh grid for at least 20 min. The mechanical sensitivities of the mice were tested using a Dynamic Plantar Aesthesiometer – DPA (Ugo Basile SRL, Italy), which can automatically detect and record paw-withdrawal thresholds. The stimuli were only applied when the mice were calm with four paws on the mesh grid but not grooming, standing with two hind legs, or sleeping. The behavioral test for mechanical sensitivity was repeated five times for each mouse and the values of pain withdrawal threshold were averaged. The behavioral test was conducted one day before surgery (day − 1) as a baseline, and 1, 2, 3, 4, 5, 6, 7, and 14 days after surgery.

### Sample collection and DNA extraction

Empty autoclaved containers were aseptically washed with 70% EtOH and sterilized in a laminar flow cabinet with UV light prior to transferring a mouse into each container. In total, 66 fecal samples were collected over 2 weeks (three mice and 11 time points per group). Fecal samples were collected right after the mice defecated and kept in 2 mL eppendorf tubes in a − 80 freezer prior until DNA extraction. The genomic DNA from the fecal samples was extracted using EasyPrep Stool Genomic DNA Kit (TOOLS, Taiwan) and qualified using a Nanodrop spectrophotometer (J&H Technology Co., Ltd., Taiwan).

### 16S rRNA amplicon preparation and Illumina sequencing

The third and fourth hypervariable regions (V3 and V4) of the 16S rRNA gene were amplified using a universal primer set. The forward primer In-341F (5′- TCGTCGGCAGCGTCAGATGTGTATAAGAGACAG-3′) and the reverse primer In-806R (5′- GTCTCGTGGGCTCGGAGATGTGTATAAGAGACAG-3′) were ligated with Illumina overhang adapters and sample-specific ten-nucleotide barcodes. Each DNA sample was PCR-amplified (Taq DNA Polymerase 2x Master Mix RED, Ampliqon, Denmark), with three replicates, under the following running conditions: initial denaturation at 95 °C for 3 min; 24 cycles of 30 s min at 95 °C, 30 s at 56 °C, 30 s at 72 °C; and a final elongation step for 10 min at 72 °C. The success of all amplicons (PCR products) were examined using 2.0% agarose gel electrophoresis. Three amplicons from each sample were pooled, isolated from the gel and purified by NucleoSpin Gel and PCR Clean-up (Macherey-Nagel, Germany). The concentration of the cleaned amplicons was qualified using a Nanodrop spectrophotometer (J&H Technology Co., Ltd., Taiwan). The amplicons were sent to the High Throughput Genomics Core, Biodiversity Research Center, Academia Sinica and processed according to the Illumina standard protocol of 16S metagenomic sequencing library preparation, and sequenced on a MiSeq platform. The Illumina fastq files were submitted to the NCBI SRA under accession number PRJNA629058.

### Amplicon sequencing data analyses

The Illumina fastq files were de-multiplexed, quality filtered, and analyzed using MOTHUR [68]. OTUs (Operational Taxonomic Units) were clustered using UPARSE [25] with a threshold of 97% pair-wise nucleotide sequence identity, and the cluster centroid for each OTU was chosen as the OTU representative sequence. OTU representative sequences were classified taxonomically using MOTHUR based on the SILVA reference database, with k-nearest neighbor consensus and Wang method, using 80% confidence as the threshold for taxonomic assignment. OTUs were determined to the genus level for these analyses. OTUs clustered at 97% that could not be assigned to a genus were assigned to a family, order, or class, and were included in the analyses. The microbial members were classified as: high-abundance (average sequencing reads more than 1% of the total sequencing reads across all time-series samples), low-abundance (average sequencing reads ranged from 0.1 to 1%), rare (average sequencing reads lower than 0.1%), core (present in all time-series samples), or non-core (present only in certain time-series samples). For alpha diversity, non-parametric Shannon’s diversity index, Inverse Simpson’s diversity index, and Chao richness were calculated using MOTHUR [68]. To increase the statistical power for the analysis of alpha diversity, we combined the time points into the following: days − 1 to 0 (6 samples), days 1–2 (6), days 3–5 (9), and days 6–14 (9). The beta diversity was illustrated using 2-dimensional non-metric multidimensional scaling (NMDS) plots of 3-dimensional data and calculated in R (vegan package [64];). Both alpha and beta diversities were performed at the genus level.

### Parameters for inferring reliable microbial interaction networks

In MetaMIS, using generalized Lotka Volterra equations, each microbial interaction network was inferred based on 177,147 (3^11^; three fecal samples and 11 time points per group) simulations. Positive or negative interactions between two nodes (i.e., OTUs at the genus level) with a permutation cutoff > 70% (124,003/177147) were considered reliable positive or negative interactions.

### Topological inferences from the microbial interaction network

Gephi was used, with each node representing an OTU (at the genus level) and each edge representing the interaction between two OTUs. The key OTUs that may play important roles in an interaction network were determined based on betweenness centrality, closeness centrality, and degree, and were positioned at the center of the network. Degree centrality measures the number of edges connecting an OTU with other OTU in the network [50]. As the number of edges connecting an OTU with other OTU increases, the degree centrality of the OTU increases. Degree is indicated by node color; a redder node corresponds to a higher value of degree. Betweenness centrality measures the number of times an OTU serves as a bridge along the shortest path between two other OTU. As the frequency of an OTU serves as a bridge increases, the betweenness centrality of the OTU increases [1]. Betweenness centrality is indicated by node size; a larger node corresponds to a higher value of betweenness centrality. Closeness centrality measures the sum of the length of the shortest path between an OTU and other OTU in the network [50]. As the closeness centrality of an OTU increases, the OTU will be positioned closer to the center of the network, thus closer to other OTU [1]. Closeness centrality is indicated by text size; larger text corresponds to a higher value of closeness centrality. The strength of the interaction between two nodes is indicated by color; the redder the edge, the more positive the interaction between its two corresponding nodes, and the bluer the edge, the more negative the interaction between its two nodes.

A rank was assigned to each OTU in the SNI and Sham groups based on the centrality score (degree, closeness and betweenness). For example, the higher the degree centrality score of an OTU, the higher the rank of the OTU based on degree centrality. Total rank is the sum of rank based on degree, closeness and betweenness centrality. A final rank was assigned to each OTU based on the total rank. The OTU with the highest final rank will be the key microbe.

### Statistical analyses

DESeq2 was used to analyze the differential relative abundance of microbes between the SNI and Sham groups (based on Benjamini-Hochberg adjusted *p*-value [55];). A permutation two sample t-test was used to analyze alpha-diversity. For beta-diversity, PERMANOVA (permutational multivariate analysis of variance) was used to analyze the microbiome data. The Shapiro–Wilk test was used to determine the normality of the proportion of positive and negative interactions in the SNI and Sham groups. The dataset of the relative positive and negative interactions in the SNI and Sham groups was not normally distributed. Hence, a Wilcoxon rank sum test was used to determine the difference in the proportions of positive or negative interactions between the SNI and Sham groups. To confirm the final rank assigned to each OTU, multiple linear regression analyses were conducted to examine the correlation between the final rank of OTU and the rank of centrality (degree, closeness, and betweeness). Statistical analyses were conducted using R [66]. *p* values < 0.05 were considered significant.

## Supplementary information

**Additional file 1: Supplemental Figure 1.** Assessment of sufficient sequencing depth by rarefaction analyses. Individual rarefaction curves for each time-series sample from the Sham (blue) or SNI (red) group. **Supplemental Figure 2:** Multiple linear regression analyses for the final ranks of microbes and centrality. A) Sham group (*p* value < 0.001; R^2^ = 0.9167), B) SNI group (p value < 0.001; R^2^ = 0.8877). **Supplemental Figure 3:** Simplified microbial interaction networks that highlight the positive and negative interactions among key microbes or probiotics within the networks. A) Microbial interaction network in the Sham group with *Oscillospira* (26) at the center; B) *Staphylococcus* (53) in the SNI group; C) *Lactobacillus* (17) in the Sham group; D) *Lactobacillus* (17) in the SNI group; E) *Bifidobacterium* (48) in the Sham group; F) *Bifidobacterium* (48) in the SNI group. **Supplemental Table 1.** Differential relative abundance between the SNI group and Sham group (*n* = 6) for each day at the genus level using DESeq2. pvalue = the average of the normalized counts, log2FoldChange = log2 fold change between the groups, lfcSE = standard error of the log2FoldChange estimate, stat = Wald statistic, pvalue = Wald test *p*-value, padj = Benjamini-Hochberg adjusted p-value. **Supplemental Table 2.1.** The ranks and values of each genus in the Sham group based on betweenness centrality, closeness centrality, and degree centrality. **Supplemental Table 2.2.** The ranks and values of each genus in the SNI group based on betweenness centrality, closeness centrality, and degree centrality.

## Data Availability

The Illumina fastq files are available in the NCBI SRA under accession number PRJNA629058. The other datasets used and/or analyzed during the current study are available from the corresponding author upon reasonable request.

## Abbreviations

gLV: Generalized Lotka–Volterra
IACUC: Institutional Animal Care and Utilization Committee
MetaMIS: Metagenomic microbial interaction simulator
OTU: Operational taxonomic unit
PCR: Polymerase chain reaction PERMANOVA Permutational multivariate analysis of variance
rRNA: Ribosomal ribonucleic acid
SNI: Spared-nerve injury
SRA: Sequence read archive
