# Correction to: A network approach to investigating the key microbes and stability of gut microbial communities in a mouse neuropathic pain model

**DOI:** 10.1186/s12866-020-02048-3

**Published:** 2020-12-28

**Authors:** Guo-Jie Brandon-Mong, Grace Tzun-Wen Shaw, Wei-Hsin Chen, Chien-Chang Chen, Daryi Wang

**Affiliations:** 1 Biodiversity Research Center, Academia Sinica, 128 Academia Road, Sec. 2, Nankang, Taipei, 11529 Taiwan; 2 Department of Life Science, National Taiwan Normal University, Taipei, Taiwan; 3 Biodiversity Program, Taiwan International Graduate Program, Academia Sinica and National Taiwan Normal University, Taipei, Taiwan; 4 Institute of Biomedical Sciences, Academia Sinica, Taipei, Taiwan; 5 Taiwan International Graduate Program in Molecular Medicine, National Yang-Ming University, Academia Sinica, 128 Academia Road, Sec. 2, Nankang, Taipei, 11529 Taiwan

**Correction to: BMC Microbiol 20, 295 (2020)**

**https://doi.org/10.1186/s12866-020-01981-7**

After publication of the original article [1], the authors have notified us that Fig. 5A was not the updated version; it was analyzed without simulations and permutation cutoff.

The correct Fig. 5A is presented below.

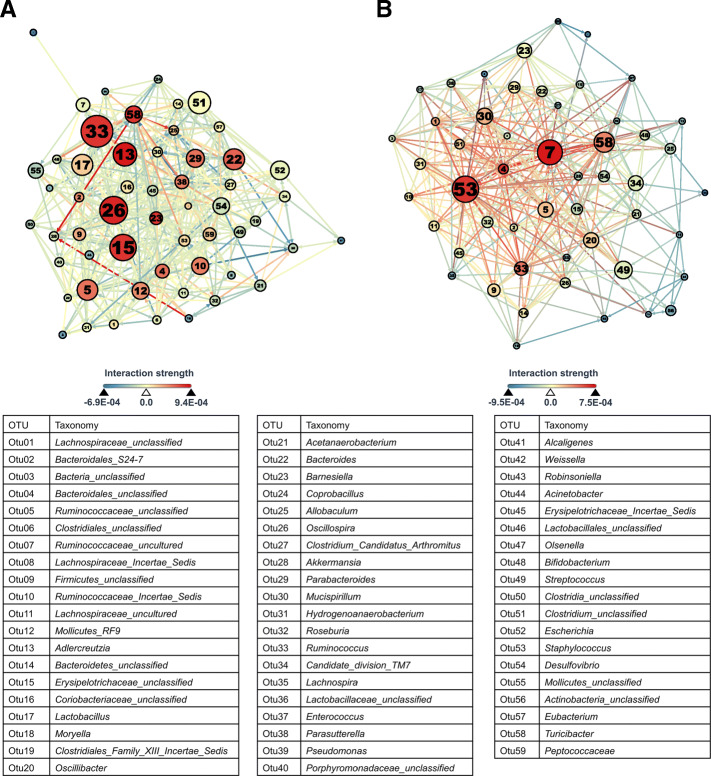



-The update of this figure does not change the results, discussion, and conclusion of the original article. We apologize for the inconvenience caused.
