# Supplementary material for: A network approach to investigating the key microbes and stability of gut microbial communities in a mouse neuropathic pain model
Source: BMC Microbiol. 2020 Sep 30;20:295. doi: 10.1186/s12866-020-01981-7 (PMC7525972; doi:10.1186/s12866-020-01981-7)
Supplement: Supplementary file 1 — Additional file 1: Supplemental Figure 1. Assessment of sufficient sequencing depth by rarefaction analyses. Individual rarefaction curves for each time-series sample from the Sham (blue) or SNI (red) group. Supplemental Figure 2: Multiple linear regression analyses for the final ranks of microbes and centrality. A) Sham group (p value < 0.001; R2 = 0.9167), B) SNI group (p value < 0.001; R2 = 0.8877). Supplemental Figure 3: Simplified microbial interaction networks that highlight the positive and negative interactions among key microbes or probiotics within the networks. A) Microbial interaction network in the Sham group with Oscillospira (26) at the center; B) Staphylococcus (53) in the SNI group; C) Lactobacillus (17) in the Sham group; D) Lactobacillus (17) in the SNI group; E) Bifidobacterium (48) in the Sham group; F) Bifidobacterium (48) in the SNI group. Supplemental Table 1. Differential relative abundance between the SNI group and Sham group (n = 6) for each day at the genus level using DESeq2. pvalue = the average of the normalized counts, log2FoldChange = log2 fold change between the groups, lfcSE = standard error of the log2FoldChange estimate, stat = Wald statistic, pvalue = Wald test p-value, padj = Benjamini-Hochberg adjusted p-value. Supplemental Table 2.1. The ranks and values of each genus in the Sham group based on betweenness centrality, closeness centrality, and degree centrality. Supplemental Table 2.2. The ranks and values of each genus in the SNI group based on betweenness centrality, closeness centrality, and degree centrality. [file 12866_2020_1981_MOESM1_ESM.docx]

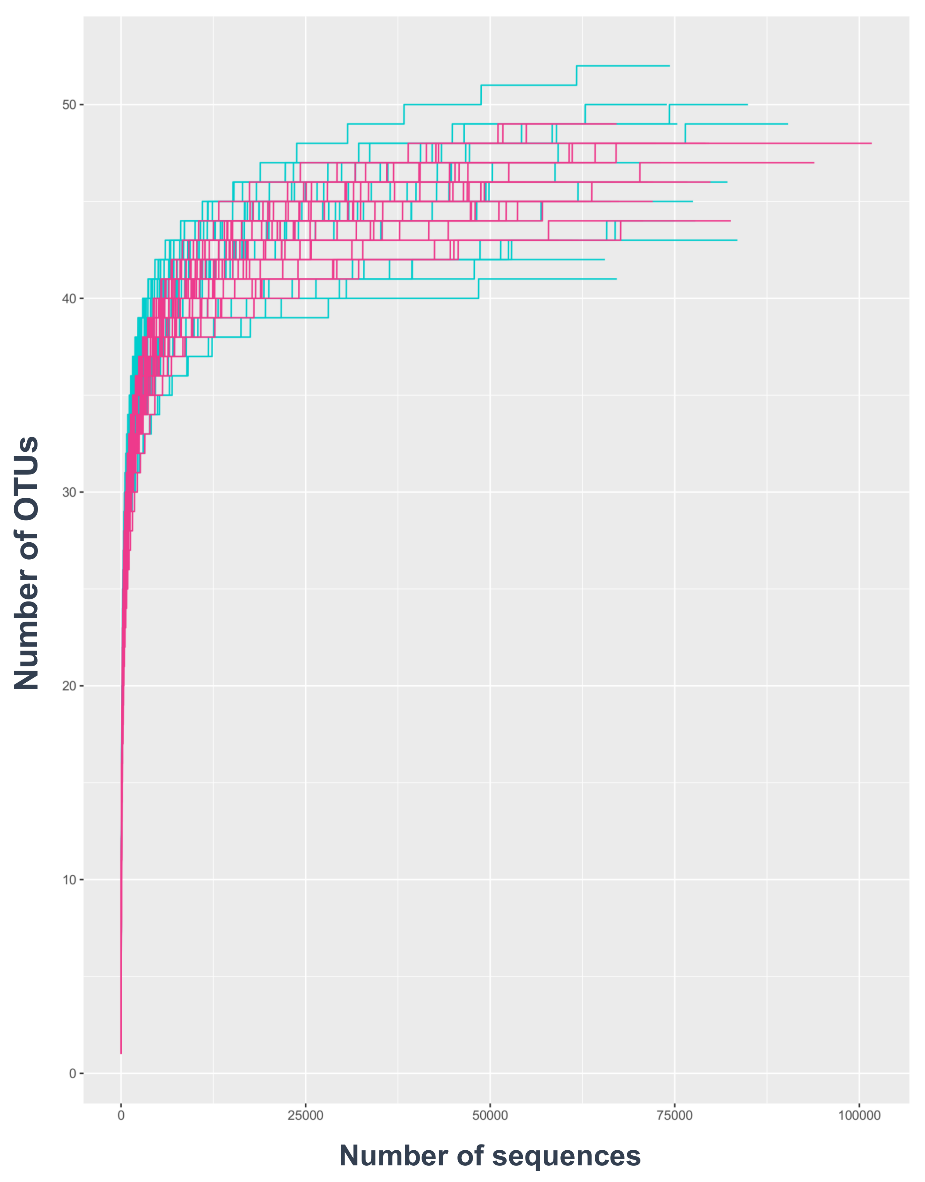


**Supplemental Figure 1.** Assessment of sufficient sequencing depth by rarefaction analyses. Individual rarefaction curves for each time-series sample from the Sham (blue) or SNI (red) group.


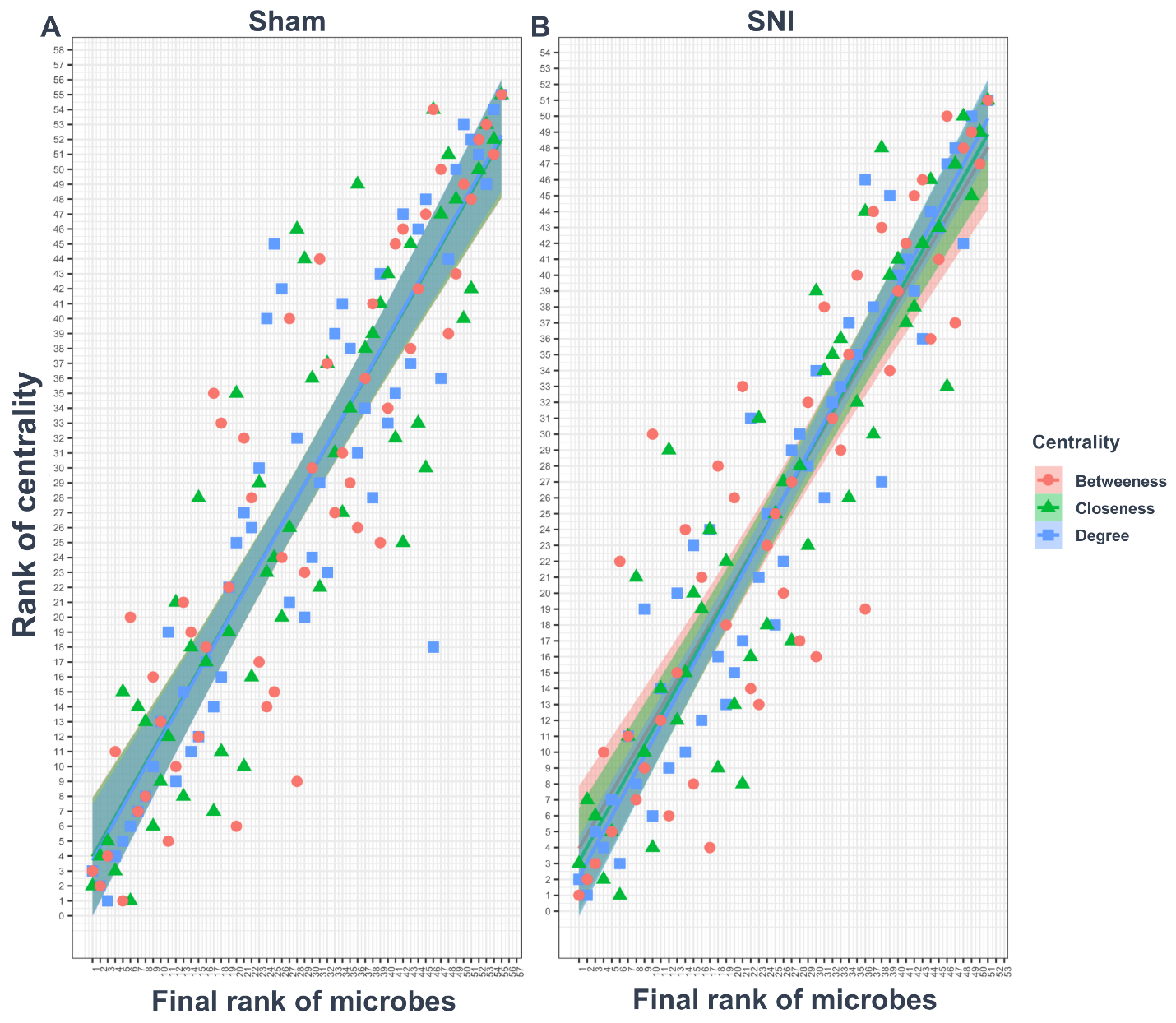


**Supplemental Figure 2:** Multiple linear regression analyses for the final ranks of microbes and centrality. A) Sham group (p value < 0.001; R^2^=0.9167), B) SNI group (p value < 0.001; R^2^=0.8877).


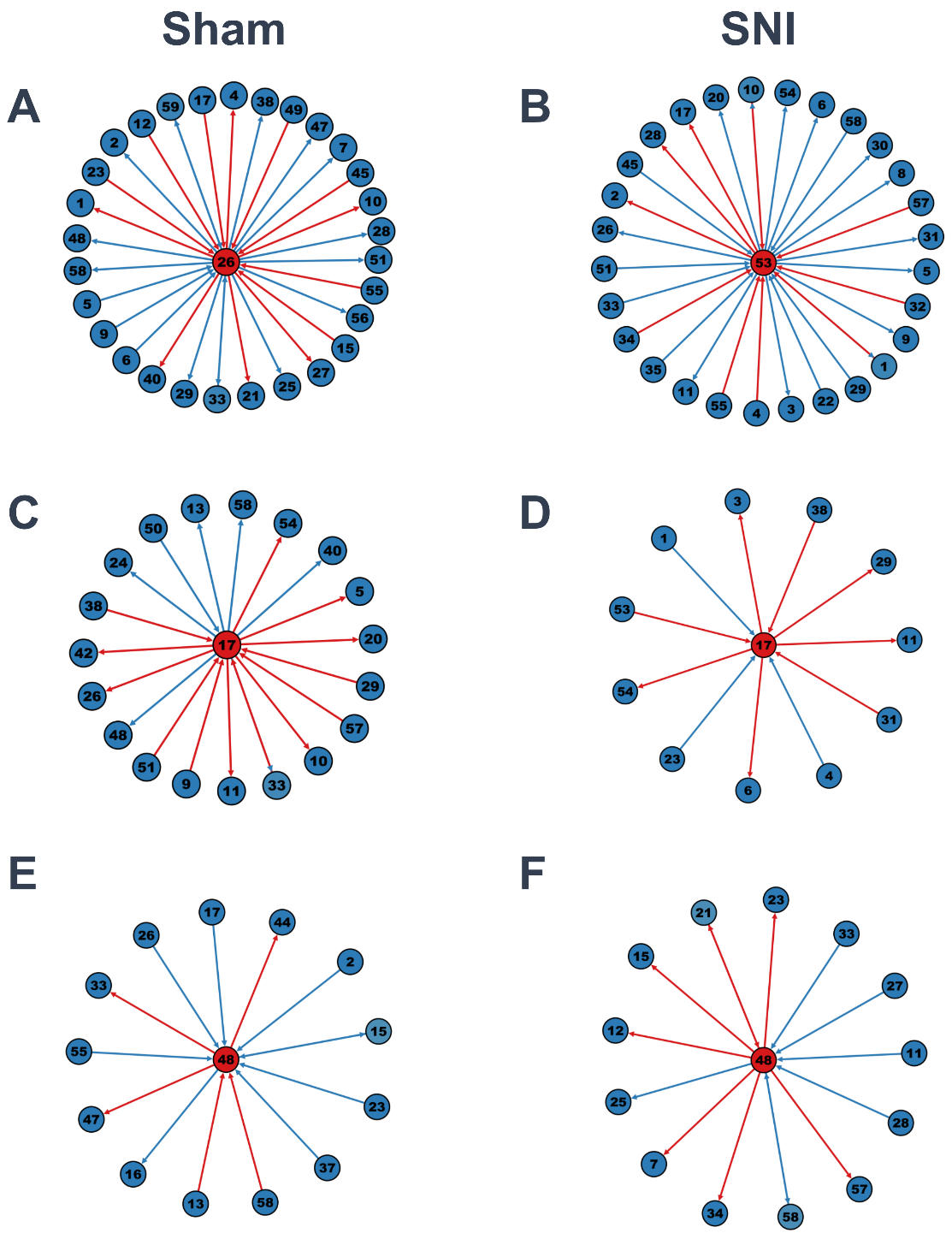


**Supplemental Figure 3:** Simplified microbial interaction networks that highlight the positive and negative interactions among key microbes or probiotics within the networks. A) Microbial interaction network in the Sham group with *Oscillospira* (26) at the center; B) *Staphylococcus* (53) in the SNI group; C) *Lactobacillus* (17) in the Sham group; D) *Lactobacillus* (17) in the SNI group; E) *Bifidobacterium* (48) in the Sham group; F) *Bifidobacterium* (48) in the SNI group.

**Supplemental Table 1.** Differential relative abundance between the SNI group and Sham group (n=6) for each day at the genus level using DESeq2. pvalue = the average of the normalized counts, log2FoldChange = log2 fold change between the groups, lfcSE = standard error of the log2FoldChange estimate, stat = Wald statistic, pvalue = Wald test p-value, padj = Benjamini-Hochberg adjusted p-value.

| Day | OTU (genus level) | baseMean | log2FoldChange | lfcSE | stat | pvalue | padj |
| --- | --- | --- | --- | --- | --- | --- | --- |
| -1-0 | *Roseburia* | 62.714 | -4.517 | 1.017 | -4.441 | 0.000 | 0.001 |
|  | *Candidate_division_TM7* | 135.684 | -2.973 | 0.831 | -3.579 | 0.000 | 0.010 |
|  | *Oscillospira* | 82.858 | -1.650 | 0.578 | -2.852 | 0.004 | 0.081 |
|  | *Oscillibacter* | 164.707 | -1.159 | 0.464 | -2.498 | 0.013 | 0.163 |
|  | *Mollicutes_unclassified* | 52.062 | -1.670 | 0.683 | -2.444 | 0.015 | 0.163 |
|  | *Turicibacter* | 884.745 | -1.581 | 0.671 | -2.355 | 0.019 | 0.173 |
|  | *Erysipelotrichaceae_Incertae_Sedis* | 453.063 | 1.347 | 0.596 | 2.260 | 0.024 | 0.191 |
|  | *Bacteroides* | 1749.943 | 0.498 | 0.270 | 1.845 | 0.065 | 0.455 |
|  | *Erysipelotrichaceae_unclassified* | 292.785 | 0.464 | 0.259 | 1.792 | 0.073 | 0.456 |
|  | *Clostridiales_unclassified* | 719.188 | 0.639 | 0.490 | 1.305 | 0.192 | 0.650 |
|  | *Firmicutes_unclassified* | 248.690 | -0.554 | 0.416 | -1.331 | 0.183 | 0.650 |
|  | *Ruminococcaceae_Incertae_Sedis* | 1428.637 | -0.457 | 0.328 | -1.394 | 0.163 | 0.650 |
|  | *Mollicutes_RF9* | 73.055 | 0.823 | 0.572 | 1.438 | 0.150 | 0.650 |
|  | *Adlercreutzia* | 613.182 | -0.479 | 0.412 | -1.162 | 0.245 | 0.650 |
|  | *Allobaculum* | 1358.112 | -1.587 | 1.108 | -1.432 | 0.152 | 0.650 |
|  | *Akkermansia* | 1153.272 | -1.148 | 0.899 | -1.276 | 0.202 | 0.650 |
|  | *Lachnospira* | 169.890 | 1.634 | 1.317 | 1.241 | 0.215 | 0.650 |
|  | *Weissella* | 0.948 | -3.349 | 2.838 | -1.180 | 0.238 | 0.650 |
|  | *Bifidobacterium* | 123.482 | -1.305 | 1.005 | -1.298 | 0.194 | 0.650 |
|  | *Staphylococcus* | 12.735 | -0.906 | 0.781 | -1.160 | 0.246 | 0.650 |
|  | *Actinobacteria_unclassified* | 7.167 | -1.060 | 0.932 | -1.137 | 0.255 | 0.650 |
|  | *Peptococcaceae_unclassified* | 33.330 | 0.821 | 0.701 | 1.170 | 0.242 | 0.650 |
|  | *Ruminococcaceae_unclassified* | 1178.460 | -0.412 | 0.379 | -1.086 | 0.277 | 0.658 |
|  | *Enterococcus* | 0.786 | -3.083 | 2.903 | -1.062 | 0.288 | 0.658 |
|  | *Parasutterella* | 591.667 | -0.551 | 0.525 | -1.050 | 0.294 | 0.658 |
|  | *Hydrogenoanaerobacterium* | 92.576 | -0.413 | 0.410 | -1.007 | 0.314 | 0.676 |
|  | *Mucispirillum* | 454.697 | -0.896 | 0.970 | -0.924 | 0.356 | 0.737 |
|  | *Bacteria_unclassified* | 350.763 | 0.349 | 0.430 | 0.812 | 0.417 | 0.805 |
|  | *Alcaligenes* | 0.533 | -1.789 | 2.151 | -0.831 | 0.406 | 0.805 |
|  | *Coriobacteriaceae_unclassified* | 756.808 | -0.297 | 0.408 | -0.728 | 0.467 | 0.810 |
|  | *Acinetobacter* | 0.531 | -1.787 | 2.601 | -0.687 | 0.492 | 0.810 |
|  | *Streptococcus* | 0.705 | 1.554 | 2.099 | 0.740 | 0.459 | 0.810 |
|  | *Escherichia* | 4.743 | 0.644 | 0.908 | 0.709 | 0.478 | 0.810 |
|  | *Eubacterium* | 94.723 | -0.499 | 0.672 | -0.742 | 0.458 | 0.810 |
|  | *Bacteroidetes_unclassified* | 37.581 | 0.453 | 0.743 | 0.610 | 0.542 | 0.853 |
|  | *Coprobacillus* | 9.001 | -0.493 | 0.820 | -0.600 | 0.548 | 0.853 |
|  | *Olsenella* | 0.463 | -1.462 | 2.638 | -0.554 | 0.580 | 0.877 |
|  | *Acetanaerobacterium* | 250.868 | 0.385 | 0.781 | 0.493 | 0.622 | 0.916 |
|  | *Lachnospiraceae_unclassified* | 13215.640 | -0.088 | 0.331 | -0.267 | 0.790 | 0.934 |
|  | *Ruminococcaceae_uncultured* | 4389.664 | 0.226 | 0.558 | 0.405 | 0.686 | 0.934 |
|  | *Lachnospiraceae_uncultured* | 481.382 | -0.155 | 0.493 | -0.314 | 0.753 | 0.934 |
|  | *Lactobacillus* | 13888.640 | 0.119 | 0.308 | 0.387 | 0.699 | 0.934 |
|  | *Barnesiella* | 4.149 | -0.231 | 0.819 | -0.282 | 0.778 | 0.934 |
|  | *Parabacteroides* | 753.440 | -0.200 | 0.490 | -0.408 | 0.683 | 0.934 |
|  | *Ruminococcus* | 230.096 | 0.205 | 0.588 | 0.349 | 0.727 | 0.934 |
|  | *Lactobacillaceae_unclassified* | 0.175 | -1.006 | 3.117 | -0.323 | 0.747 | 0.934 |
|  | *Clostridia_unclassified* | 0.669 | -0.720 | 2.162 | -0.333 | 0.739 | 0.934 |
|  | *Desulfovibrio* | 1794.303 | -0.144 | 0.568 | -0.253 | 0.800 | 0.934 |
|  | *Bacteroidales_unclassified* | 25769.710 | 0.029 | 0.221 | 0.129 | 0.897 | 0.954 |
|  | *Lachnospiraceae_Incertae_Sedis* | 1766.359 | 0.041 | 0.339 | 0.122 | 0.903 | 0.954 |
|  | *Moryella* | 699.780 | -0.149 | 1.058 | -0.141 | 0.888 | 0.954 |
|  | *Porphyromonadaceae_unclassified* | 0.105 | 0.437 | 3.117 | 0.140 | 0.889 | 0.954 |
|  | *Clostridium_unclassified* | 8.129 | -0.141 | 1.116 | -0.127 | 0.899 | 0.954 |
|  | *Clostridiales_Family_XIII_Incertae_Sedis* | 62.580 | 0.016 | 0.288 | 0.054 | 0.957 | 0.992 |
|  | *Bacteroidales_S24-7* | 25916.790 | -0.001 | 0.267 | -0.003 | 0.998 | 0.998 |
|  | *Clostridium_Candidatus_Arthromitus* | 882.264 | 0.002 | 0.489 | 0.005 | 0.996 | 0.998 |
|  | *Pseudomonas* | 0.000 | NA | NA | NA | NA | NA |
|  | *Robinsoniella* | 0.000 | NA | NA | NA | NA | NA |
|  | *Lactobacillales_unclassified* | 0.000 | NA | NA | NA | NA | NA |
| 1-2 | *Roseburia* | 203.951 | -5.483 | 0.918 | -5.974 | 0.000 | 0.000 |
|  | *Erysipelotrichaceae_Incertae_Sedis* | 188.432 | 1.492 | 0.417 | 3.579 | 0.000 | 0.009 |
|  | *Mollicutes_unclassified* | 61.731 | -1.897 | 0.558 | -3.399 | 0.001 | 0.012 |
|  | *Peptococcaceae_unclassified* | 23.395 | 1.658 | 0.503 | 3.298 | 0.001 | 0.013 |
|  | *Oscillospira* | 208.386 | -1.402 | 0.506 | -2.769 | 0.006 | 0.062 |
|  | *Ruminococcaceae_uncultured* | 3868.189 | 0.795 | 0.402 | 1.978 | 0.048 | 0.293 |
|  | *Ruminococcaceae_Incertae_Sedis* | 2438.537 | -0.614 | 0.296 | -2.073 | 0.038 | 0.293 |
|  | *Bacteroides* | 957.078 | 0.502 | 0.252 | 1.988 | 0.047 | 0.293 |
|  | *Turicibacter* | 60.716 | -1.359 | 0.645 | -2.108 | 0.035 | 0.293 |
|  | *Lachnospiraceae_unclassified* | 19571.050 | -0.449 | 0.243 | -1.844 | 0.065 | 0.358 |
|  | *Mollicutes_RF9* | 75.006 | 0.792 | 0.448 | 1.770 | 0.077 | 0.384 |
|  | *Erysipelotrichaceae_unclassified* | 378.665 | 0.525 | 0.326 | 1.611 | 0.107 | 0.459 |
|  | *Moryella* | 826.457 | -1.176 | 0.749 | -1.569 | 0.117 | 0.459 |
|  | *Oscillibacter* | 315.479 | -0.781 | 0.498 | -1.569 | 0.117 | 0.459 |
|  | *Candidate_division_TM7* | 108.298 | -1.189 | 0.787 | -1.510 | 0.131 | 0.480 |
|  | *Hydrogenoanaerobacterium* | 148.623 | -0.573 | 0.395 | -1.451 | 0.147 | 0.505 |
|  | *Ruminococcus* | 80.906 | 0.618 | 0.526 | 1.175 | 0.240 | 0.690 |
|  | *Lachnospira* | 270.374 | 1.581 | 1.370 | 1.154 | 0.248 | 0.690 |
|  | *Bifidobacterium* | 8.855 | 1.289 | 1.072 | 1.203 | 0.229 | 0.690 |
|  | *Staphylococcus* | 0.722 | -2.197 | 1.913 | -1.148 | 0.251 | 0.690 |
|  | *Barnesiella* | 10.902 | -0.461 | 0.430 | -1.071 | 0.284 | 0.744 |
|  | *Parabacteroides* | 374.679 | 0.411 | 0.433 | 0.949 | 0.343 | 0.820 |
|  | *Mucispirillum* | 1337.309 | 0.712 | 0.736 | 0.968 | 0.333 | 0.820 |
|  | *Clostridiales_Family_XIII_Incertae_Sedis* | 45.123 | 0.218 | 0.258 | 0.847 | 0.397 | 0.910 |
|  | *Bacteroidales_S24-7* | 20312.160 | 0.157 | 0.345 | 0.457 | 0.648 | 0.932 |
|  | *Bacteria_unclassified* | 514.182 | 0.131 | 0.314 | 0.418 | 0.676 | 0.932 |
|  | *Bacteroidales_unclassified* | 27894.100 | -0.202 | 0.336 | -0.600 | 0.548 | 0.932 |
|  | *Ruminococcaceae_unclassified* | 1991.674 | -0.140 | 0.401 | -0.348 | 0.728 | 0.932 |
|  | *Clostridiales_unclassified* | 774.569 | -0.059 | 0.345 | -0.170 | 0.865 | 0.932 |
|  | *Lachnospiraceae_Incertae_Sedis* | 2399.996 | -0.076 | 0.267 | -0.286 | 0.775 | 0.932 |
|  | *Lachnospiraceae_uncultured* | 630.628 | -0.290 | 0.442 | -0.656 | 0.512 | 0.932 |
|  | *Adlercreutzia* | 450.881 | 0.061 | 0.479 | 0.128 | 0.898 | 0.932 |
|  | *Bacteroidetes_unclassified* | 19.308 | 0.347 | 0.487 | 0.712 | 0.476 | 0.932 |
|  | *Coriobacteriaceae_unclassified* | 695.916 | 0.063 | 0.433 | 0.145 | 0.885 | 0.932 |
|  | *Lactobacillus* | 9737.255 | 0.207 | 0.470 | 0.440 | 0.660 | 0.932 |
|  | *Acetanaerobacterium* | 331.229 | -0.174 | 0.660 | -0.264 | 0.792 | 0.932 |
|  | *Coprobacillus* | 6.823 | 0.539 | 0.794 | 0.678 | 0.498 | 0.932 |
|  | *Allobaculum* | 18.034 | 0.480 | 0.968 | 0.496 | 0.620 | 0.932 |
|  | *Clostridium_Candidatus_Arthromitus* | 761.604 | 0.162 | 0.510 | 0.319 | 0.750 | 0.932 |
|  | *Akkermansia* | 104.755 | -0.477 | 0.751 | -0.635 | 0.526 | 0.932 |
|  | *Lactobacillaceae_unclassified* | 0.091 | 0.546 | 3.041 | 0.180 | 0.857 | 0.932 |
|  | *Enterococcus* | 1.214 | -0.879 | 1.869 | -0.471 | 0.638 | 0.932 |
|  | *Parasutterella* | 94.022 | -0.141 | 0.449 | -0.314 | 0.753 | 0.932 |
|  | *Porphyromonadaceae_unclassified* | 0.458 | -0.461 | 1.966 | -0.234 | 0.815 | 0.932 |
|  | *Alcaligenes* | 0.244 | 1.205 | 3.039 | 0.397 | 0.692 | 0.932 |
|  | *Weissella* | 0.398 | 0.619 | 3.037 | 0.204 | 0.839 | 0.932 |
|  | *Acinetobacter* | 0.341 | 1.889 | 2.813 | 0.672 | 0.502 | 0.932 |
|  | *Streptococcus* | 2.049 | 0.150 | 1.137 | 0.132 | 0.895 | 0.932 |
|  | *Clostridia_unclassified* | 1.147 | 0.808 | 1.558 | 0.518 | 0.604 | 0.932 |
|  | *Clostridium_unclassified* | 0.100 | -0.736 | 3.041 | -0.242 | 0.809 | 0.932 |
|  | *Escherichia* | 3.360 | 0.325 | 0.899 | 0.361 | 0.718 | 0.932 |
|  | *Actinobacteria_unclassified* | 3.450 | 0.594 | 1.120 | 0.530 | 0.596 | 0.932 |
|  | *Eubacterium* | 39.716 | 0.280 | 0.361 | 0.775 | 0.438 | 0.932 |
|  | *Firmicutes_unclassified* | 347.906 | 0.027 | 0.378 | 0.072 | 0.942 | 0.960 |
|  | *Desulfovibrio* | 2233.314 | -0.009 | 0.340 | -0.027 | 0.978 | 0.978 |
|  | *Pseudomonas* | 0.000 | NA | NA | NA | NA | NA |
|  | *Robinsoniella* | 0.000 | NA | NA | NA | NA | NA |
|  | *Lactobacillales_unclassified* | 0.000 | NA | NA | NA | NA | NA |
|  | *Olsenella* | 0.000 | NA | NA | NA | NA | NA |
| 3-5 | *Turicibacter* | 631.411 | -6.701 | 1.000 | -6.702 | 0.000 | 0.000 |
|  | *Roseburia* | 140.210 | -2.486 | 0.655 | -3.797 | 0.000 | 0.004 |
|  | *Mollicutes_unclassified* | 98.112 | -2.461 | 0.681 | -3.615 | 0.000 | 0.005 |
|  | *Allobaculum* | 25.422 | -3.424 | 1.246 | -2.748 | 0.006 | 0.081 |
|  | *Akkermansia* | 56.738 | -2.521 | 0.954 | -2.641 | 0.008 | 0.089 |
|  | *Erysipelotrichaceae_unclassified* | 265.972 | 0.549 | 0.217 | 2.532 | 0.011 | 0.102 |
|  | *Ruminococcaceae_uncultured* | 4061.053 | -0.730 | 0.378 | -1.932 | 0.053 | 0.412 |
|  | *Clostridiales_unclassified* | 1247.357 | -0.728 | 0.456 | -1.595 | 0.111 | 0.498 |
|  | *Mollicutes_RF9* | 76.930 | -0.891 | 0.504 | -1.768 | 0.077 | 0.498 |
|  | *Adlercreutzia* | 525.720 | -0.562 | 0.343 | -1.636 | 0.102 | 0.498 |
|  | *Lachnospira* | 65.702 | 2.331 | 1.389 | 1.678 | 0.093 | 0.498 |
|  | *Escherichia* | 1.247 | 2.143 | 1.330 | 1.611 | 0.107 | 0.498 |
|  | *Bacteroides* | 699.758 | -0.504 | 0.386 | -1.306 | 0.191 | 0.678 |
|  | *Oscillospira* | 186.395 | -0.777 | 0.583 | -1.334 | 0.182 | 0.678 |
|  | *Bifidobacterium* | 19.814 | -1.457 | 1.139 | -1.279 | 0.201 | 0.678 |
|  | *Actinobacteria_unclassified* | 3.566 | -0.942 | 0.709 | -1.328 | 0.184 | 0.678 |
|  | *Clostridia_unclassified* | 1.284 | -0.984 | 0.903 | -1.090 | 0.276 | 0.876 |
|  | *Lachnospiraceae_Incertae_Sedis* | 2123.227 | 0.307 | 0.317 | 0.971 | 0.332 | 0.943 |
|  | *Eubacterium* | 38.671 | 0.356 | 0.365 | 0.977 | 0.329 | 0.943 |
|  | *Bacteroidales_unclassified* | 26872.280 | -0.229 | 0.326 | -0.702 | 0.483 | 0.946 |
|  | *Ruminococcaceae_unclassified* | 2256.148 | 0.250 | 0.318 | 0.787 | 0.431 | 0.946 |
|  | *Clostridiales_Family_XIII_Incertae_Sedis* | 44.092 | 0.165 | 0.222 | 0.742 | 0.458 | 0.946 |
|  | *Oscillibacter* | 394.616 | -0.226 | 0.327 | -0.689 | 0.491 | 0.946 |
|  | *Barnesiella* | 10.626 | 0.315 | 0.351 | 0.898 | 0.369 | 0.946 |
|  | *Coprobacillus* | 9.738 | -0.494 | 0.594 | -0.831 | 0.406 | 0.946 |
|  | *Mucispirillum* | 1154.058 | 0.797 | 0.880 | 0.906 | 0.365 | 0.946 |
|  | *Candidate_division_TM7* | 148.860 | -0.623 | 0.875 | -0.712 | 0.476 | 0.946 |
|  | *Streptococcus* | 0.754 | 1.198 | 1.570 | 0.763 | 0.445 | 0.946 |
|  | *Lachnospiraceae_unclassified* | 19790.400 | -0.039 | 0.272 | -0.144 | 0.886 | 0.965 |
|  | *Bacteroidales_S24-7* | 21115.510 | 0.019 | 0.276 | 0.068 | 0.946 | 0.965 |
|  | *Bacteria_unclassified* | 623.696 | -0.112 | 0.385 | -0.290 | 0.772 | 0.965 |
|  | *Firmicutes_unclassified* | 409.388 | 0.211 | 0.442 | 0.479 | 0.632 | 0.965 |
|  | *Ruminococcaceae_Incertae_Sedis* | 2726.318 | 0.041 | 0.321 | 0.127 | 0.899 | 0.965 |
|  | *Lachnospiraceae_uncultured* | 655.510 | 0.100 | 0.439 | 0.228 | 0.820 | 0.965 |
|  | *Bacteroidetes_unclassified* | 18.732 | 0.235 | 0.505 | 0.464 | 0.642 | 0.965 |
|  | *Coriobacteriaceae_unclassified* | 683.513 | -0.089 | 0.294 | -0.303 | 0.762 | 0.965 |
|  | *Lactobacillus* | 6887.151 | 0.094 | 0.437 | 0.215 | 0.830 | 0.965 |
|  | *Moryella* | 1242.370 | 0.439 | 0.776 | 0.565 | 0.572 | 0.965 |
|  | *Acetanaerobacterium* | 369.647 | 0.087 | 0.514 | 0.169 | 0.866 | 0.965 |
|  | *Clostridium_Candidatus_Arthromitus* | 408.850 | -0.084 | 0.412 | -0.204 | 0.838 | 0.965 |
|  | *Parabacteroides* | 253.765 | -0.190 | 0.431 | -0.440 | 0.660 | 0.965 |
|  | *Hydrogenoanaerobacterium* | 167.662 | -0.037 | 0.409 | -0.090 | 0.929 | 0.965 |
|  | *Ruminococcus* | 77.888 | -0.175 | 0.628 | -0.279 | 0.780 | 0.965 |
|  | *Lactobacillaceae_unclassified* | 0.137 | -0.760 | 3.041 | -0.250 | 0.803 | 0.965 |
|  | *Enterococcus* | 0.106 | -0.760 | 3.041 | -0.250 | 0.803 | 0.965 |
|  | *Parasutterella* | 122.634 | -0.030 | 0.687 | -0.044 | 0.965 | 0.965 |
|  | *Porphyromonadaceae_unclassified* | 0.124 | 0.522 | 3.041 | 0.172 | 0.864 | 0.965 |
|  | *Weissella* | 0.052 | -0.440 | 3.041 | -0.145 | 0.885 | 0.965 |
|  | *Robinsoniella* | 0.048 | 0.201 | 3.041 | 0.066 | 0.947 | 0.965 |
|  | *Acinetobacter* | 0.372 | -0.431 | 1.729 | -0.250 | 0.803 | 0.965 |
|  | *Erysipelotrichaceae_Incertae_Sedis* | 122.251 | 0.194 | 0.478 | 0.407 | 0.684 | 0.965 |
|  | *Staphylococcus* | 0.133 | 0.522 | 3.041 | 0.172 | 0.864 | 0.965 |
|  | *Desulfovibrio* | 2637.107 | 0.019 | 0.373 | 0.052 | 0.959 | 0.965 |
|  | *Peptococcaceae_unclassified* | 26.871 | -0.083 | 0.501 | -0.165 | 0.869 | 0.965 |
|  | *Pseudomonas* | 0.000 | NA | NA | NA | NA | NA |
|  | *Alcaligenes* | 0.000 | NA | NA | NA | NA | NA |
|  | *Lactobacillales_unclassified* | 0.000 | NA | NA | NA | NA | NA |
|  | *Olsenella* | 0.000 | NA | NA | NA | NA | NA |
|  | *Clostridium_unclassified* | 0.000 | NA | NA | NA | NA | NA |
| 6-14 | *Turicibacter* | 2429.798 | -8.123 | 0.468 | -17.372 | 0.000 | 0.000 |
|  | *Allobaculum* | 986.518 | -7.646 | 0.787 | -9.710 | 0.000 | 0.000 |
|  | *Bifidobacterium* | 244.424 | -5.224 | 0.843 | -6.200 | 0.000 | 0.000 |
|  | *Akkermansia* | 1560.327 | -4.094 | 0.885 | -4.626 | 0.000 | 0.000 |
|  | *Roseburia* | 178.999 | -2.746 | 0.657 | -4.179 | 0.000 | 0.000 |
|  | *Mollicutes_unclassified* | 92.382 | -1.838 | 0.557 | -3.299 | 0.001 | 0.009 |
|  | *Lactobacillus* | 10075.430 | 1.170 | 0.371 | 3.154 | 0.002 | 0.013 |
|  | *Actinobacteria_unclassified* | 10.271 | -1.951 | 0.692 | -2.817 | 0.005 | 0.033 |
|  | *Clostridium_Candidatus_Arthromitus* | 430.891 | 1.599 | 0.625 | 2.557 | 0.011 | 0.064 |
|  | *Peptococcaceae_unclassified* | 40.375 | -1.070 | 0.440 | -2.435 | 0.015 | 0.082 |
|  | *Olsenella* | 4.024 | -5.444 | 2.403 | -2.266 | 0.023 | 0.117 |
|  | *Clostridium_unclassified* | 1.213 | -3.702 | 1.678 | -2.206 | 0.027 | 0.125 |
|  | *Parasutterella* | 335.168 | -0.979 | 0.491 | -1.992 | 0.046 | 0.196 |
|  | *Erysipelotrichaceae_unclassified* | 230.802 | 0.478 | 0.256 | 1.867 | 0.062 | 0.239 |
|  | *Coriobacteriaceae_unclassified* | 708.598 | -0.491 | 0.266 | -1.844 | 0.065 | 0.239 |
|  | *Ruminococcaceae_Incertae_Sedis* | 2045.811 | 0.500 | 0.276 | 1.813 | 0.070 | 0.240 |
|  | *Candidate_division_TM7* | 85.685 | -1.409 | 0.796 | -1.769 | 0.077 | 0.249 |
|  | *Erysipelotrichaceae_Incertae_Sedis* | 234.722 | -0.777 | 0.448 | -1.735 | 0.083 | 0.253 |
|  | *Bacteroidales_unclassified* | 22840.610 | 0.463 | 0.274 | 1.687 | 0.092 | 0.265 |
|  | *Bacteroides* | 899.207 | -0.345 | 0.208 | -1.659 | 0.097 | 0.267 |
|  | *Ruminococcaceae_unclassified* | 1679.435 | 0.331 | 0.223 | 1.486 | 0.137 | 0.343 |
|  | *Eubacterium* | 72.790 | 0.593 | 0.394 | 1.503 | 0.133 | 0.343 |
|  | *Hydrogenoanaerobacterium* | 121.319 | 0.551 | 0.416 | 1.326 | 0.185 | 0.442 |
|  | *Ruminococcaceae_uncultured* | 4636.824 | -0.542 | 0.432 | -1.255 | 0.209 | 0.461 |
|  | *Escherichia* | 2.328 | 2.116 | 1.673 | 1.265 | 0.206 | 0.461 |
|  | *Clostridiales_unclassified* | 785.678 | -0.445 | 0.370 | -1.203 | 0.229 | 0.484 |
|  | *Oscillospira* | 90.541 | 0.598 | 0.508 | 1.176 | 0.240 | 0.488 |
|  | *Mucispirillum* | 607.549 | 0.810 | 0.714 | 1.134 | 0.257 | 0.492 |
|  | *Clostridia_unclassified* | 1.406 | -1.466 | 1.318 | -1.112 | 0.266 | 0.492 |
|  | *Desulfovibrio* | 2047.329 | -0.477 | 0.432 | -1.106 | 0.269 | 0.492 |
|  | *Lachnospira* | 41.722 | 0.986 | 0.962 | 1.025 | 0.306 | 0.542 |
|  | *Barnesiella* | 7.849 | -0.480 | 0.496 | -0.968 | 0.333 | 0.572 |
|  | *Parabacteroides* | 311.123 | -0.377 | 0.399 | -0.945 | 0.345 | 0.575 |
|  | *Adlercreutzia* | 680.933 | -0.393 | 0.427 | -0.921 | 0.357 | 0.578 |
|  | *Acetanaerobacterium* | 324.409 | 0.452 | 0.528 | 0.855 | 0.393 | 0.617 |
|  | *Bacteroidetes_unclassified* | 23.406 | 0.406 | 0.552 | 0.735 | 0.462 | 0.706 |
|  | *Oscillibacter* | 284.625 | -0.246 | 0.395 | -0.624 | 0.532 | 0.785 |
|  | *Streptococcus* | 0.790 | 1.162 | 1.907 | 0.610 | 0.542 | 0.785 |
|  | *Mollicutes_RF9* | 58.116 | 0.319 | 0.546 | 0.583 | 0.560 | 0.789 |
|  | *Enterococcus* | 0.602 | 1.683 | 3.007 | 0.560 | 0.576 | 0.791 |
|  | *Lachnospiraceae_unclassified* | 15650.410 | -0.158 | 0.305 | -0.517 | 0.605 | 0.812 |
|  | *Lachnospiraceae_uncultured* | 450.361 | 0.177 | 0.390 | 0.455 | 0.649 | 0.850 |
|  | *Lachnospiraceae_Incertae_Sedis* | 2012.209 | 0.073 | 0.281 | 0.261 | 0.794 | 0.975 |
|  | *Firmicutes_unclassified* | 316.789 | 0.065 | 0.345 | 0.188 | 0.851 | 0.975 |
|  | *Moryella* | 1248.662 | 0.216 | 0.755 | 0.287 | 0.774 | 0.975 |
|  | *Ruminococcus* | 198.702 | 0.143 | 0.689 | 0.207 | 0.836 | 0.975 |
|  | *Pseudomonas* | 0.110 | 0.630 | 3.041 | 0.207 | 0.836 | 0.975 |
|  | *Weissella* | 0.092 | 0.630 | 3.041 | 0.207 | 0.836 | 0.975 |
|  | *Bacteroidales_S24-7* | 27833.510 | -0.038 | 0.292 | -0.130 | 0.897 | 0.990 |
|  | *Bacteria_unclassified* | 490.248 | -0.047 | 0.374 | -0.126 | 0.900 | 0.990 |
|  | *Clostridiales_Family_XIII_Incertae_Sedis* | 58.296 | 0.031 | 0.319 | 0.098 | 0.922 | 0.993 |
|  | *Coprobacillus* | 16.427 | -0.061 | 0.798 | -0.077 | 0.939 | 0.993 |
|  | *Acinetobacter* | 0.402 | -0.061 | 2.373 | -0.026 | 0.979 | 0.997 |
|  | *Lactobacillales_unclassified* | 0.200 | -0.011 | 3.041 | -0.004 | 0.997 | 0.997 |
|  | *Staphylococcus* | 10.290 | 0.034 | 1.048 | 0.032 | 0.974 | 0.997 |
|  | *Lactobacillaceae_unclassified* | 0.000 | NA | NA | NA | NA | NA |
|  | *Porphyromonadaceae_unclassified* | 0.000 | NA | NA | NA | NA | NA |
|  | *Alcaligenes* | 0.000 | NA | NA | NA | NA | NA |
|  | *Robinsoniella* | 0.000 | NA | NA | NA | NA | NA |

**Supplemental Table 2.1.** The ranks and values of each genus in the Sham group based on betweenness centrality, closeness centrality, and degree centrality.

| OTU | Genus | Degree centrality score | Rank based on degree centrality  score | Closeness  centrality score | Rank based on closeness  centrality  score | Betweeness centrality score | Rank based on betweeness  centrality  score | Total  rank | Final rank |
| --- | --- | --- | --- | --- | --- | --- | --- | --- | --- |
| 15 | *Erysipelotrichaceae_unclassified* | 30 | 3 | 0.635294 | 2 | 0.070859 | 3 | 8 | 1 |
| 26 | *Oscillospira* | 31 | 2 | 0.606742 | 4 | 0.075661 | 2 | 8 | 1 |
| 13 | *Adlercreutzia* | 32 | 1 | 0.593407 | 5 | 0.060596 | 4 | 10 | 2 |
| 58 | *Turicibacter* | 30 | 4 | 0.613636 | 3 | 0.03778 | 11 | 18 | 3 |
| 33 | *Ruminococcus* | 30 | 5 | 0.514286 | 15 | 0.090011 | 1 | 21 | 4 |
| 23 | *Barnesiella* | 30 | 6 | 0.658537 | 1 | 0.022513 | 20 | 27 | 5 |
| 22 | *Bacteroides* | 27 | 7 | 0.524272 | 14 | 0.051845 | 7 | 28 | 6 |
| 5 | *Ruminococcaceae_unclassified* | 26 | 8 | 0.529412 | 13 | 0.050517 | 8 | 29 | 7 |
| 4 | *Bacteroidales_unclassified* | 26 | 10 | 0.593407 | 6 | 0.026636 | 16 | 32 | 8 |
| 12 | *Mollicutes_RF9* | 24 | 13 | 0.54 | 9 | 0.037608 | 13 | 35 | 9 |
| 17 | *Lactobacillus* | 20 | 19 | 0.534653 | 12 | 0.051958 | 5 | 36 | 10 |
| 29 | *Parabacteroides* | 26 | 9 | 0.486486 | 21 | 0.04131 | 10 | 40 | 11 |
| 9 | *Firmicutes_unclassified* | 22 | 15 | 0.568421 | 8 | 0.021304 | 21 | 44 | 12 |
| 38 | *Parasutterella* | 25 | 11 | 0.495413 | 18 | 0.025906 | 19 | 48 | 13 |
| 10 | *Ruminococcaceae_Incertae_Sedis* | 25 | 12 | 0.461538 | 28 | 0.03788 | 12 | 52 | 14 |
| 59 | *Peptococcaceae_unclassified* | 20 | 17 | 0.504673 | 17 | 0.023233 | 18 | 52 | 15 |
| 2 | *Bacteroidales_S24-7* | 24 | 14 | 0.586957 | 7 | 0.010977 | 35 | 56 | 16 |
| 25 | *Allobaculum* | 20 | 16 | 0.534653 | 11 | 0.011336 | 33 | 60 | 17 |
| 16 | *Coriobacteriaceae_;unclassified* | 18 | 22 | 0.495413 | 19 | 0.021189 | 22 | 63 | 18 |
| 51 | *Clostridium_unclassified* | 16 | 25 | 0.421875 | 35 | 0.056591 | 6 | 66 | 19 |
| 30 | *Mucispirillum* | 16 | 27 | 0.54 | 10 | 0.012288 | 32 | 69 | 20 |
| 27 | *Clostridium_Candidatus_Arthromitus* | 16 | 26 | 0.509434 | 16 | 0.014025 | 28 | 70 | 21 |
| 7 | *Ruminococcaceae_uncultured* | 15 | 30 | 0.45 | 29 | 0.026189 | 17 | 76 | 22 |
| 54 | *Desulfovibrio* | 13 | 40 | 0.473684 | 23 | 0.036011 | 14 | 77 | 23 |
| 55 | *Mollicutes_unclassified* | 11 | 45 | 0.473684 | 24 | 0.031616 | 15 | 84 | 24 |
| 49 | *Streptococcus* | 13 | 42 | 0.490909 | 20 | 0.016603 | 24 | 86 | 25 |
| 1 | *Lachnospiraceae_unclassified* | 18 | 21 | 0.465517 | 26 | 0.009072 | 40 | 87 | 26 |
| 52 | *Escherichia* | 15 | 32 | 0.369863 | 46 | 0.043644 | 9 | 87 | 27 |
| 53 | *Staphylococcus* | 19 | 20 | 0.380282 | 44 | 0.016718 | 23 | 87 | 28 |
| 57 | *Eubacterium* | 17 | 24 | 0.421875 | 36 | 0.013173 | 30 | 90 | 29 |
| 6 | *Clostridiales_unclassified* | 15 | 29 | 0.482143 | 22 | 0.007432 | 44 | 95 | 30 |
| 14 | *Bacteroidetes_unclassified* | 17 | 23 | 0.415385 | 37 | 0.01002 | 37 | 97 | 31 |
| 45 | *Erysipelotrichaceae_Incertae_Sedis* | 13 | 39 | 0.439024 | 31 | 0.014139 | 27 | 97 | 32 |
| 19 | *Clostridiales_Family_XIII_Incertae_Sedis* | 13 | 41 | 0.465517 | 27 | 0.012976 | 31 | 99 | 33 |
| 32 | *Roseburia* | 13 | 38 | 0.421875 | 34 | 0.013423 | 29 | 101 | 34 |
| 34 | *Candidate_division_TM7* | 15 | 31 | 0.312139 | 49 | 0.015425 | 26 | 106 | 35 |
| 28 | *Akkermansia* | 14 | 34 | 0.409091 | 38 | 0.0103 | 36 | 108 | 37 |
| 31 | *Hydrogenoanaerobacterium* | 15 | 28 | 0.397059 | 39 | 0.008491 | 41 | 108 | 38 |
| 21 | *Acetanaerobacterium* | 12 | 43 | 0.394161 | 41 | 0.017137 | 25 | 109 | 40 |
| 11 | *Lachnospiraceae_uncultured* | 14 | 33 | 0.388489 | 43 | 0.011192 | 34 | 110 | 41 |
| 48 | *Bifidobacterium* | 14 | 35 | 0.439024 | 32 | 0.007303 | 45 | 112 | 42 |
| 24 | *Coprobacillus* | 11 | 47 | 0.473684 | 25 | 0.00496 | 46 | 118 | 44 |
| 40 | *Porphyromonadaceae_unclassified* | 13 | 37 | 0.377622 | 45 | 0.009755 | 38 | 120 | 45 |
| 50 | *Clostridia_unclassified* | 11 | 46 | 0.425197 | 33 | 0.007948 | 42 | 121 | 47 |
| 8 | *Lachnospiraceae_Incertae_Sedis* | 9 | 48 | 0.442623 | 30 | 0.004331 | 47 | 125 | 48 |
| 47 | *Olsenella* | 20 | 18 | 0 | 54 | 0 | 54 | 126 | 49 |
| 20 | *Oscillibacter* | 13 | 36 | 0.348387 | 47 | 0.002927 | 50 | 133 | 50 |
| 35 | *Lachnospira* | 12 | 44 | 0.278351 | 51 | 0.009703 | 39 | 134 | 51 |
| 44 | *Acinetobacter* | 8 | 50 | 0.325301 | 48 | 0.007594 | 43 | 141 | 52 |
| 3 | *Bacteria_unclassified* | 6 | 53 | 0.394161 | 40 | 0.003004 | 49 | 142 | 53 |
| 18 | *Moryella* | 7 | 52 | 0.391304 | 42 | 0.003227 | 48 | 142 | 54 |
| 56 | *Actinobacteria_unclassified* | 8 | 51 | 0.301676 | 50 | 0.000426 | 52 | 153 | 55 |
| 42 | *Weissella* | 8 | 49 | 0 | 53 | 0 | 53 | 155 | 56 |
| 37 | *Enterococcus* | 5 | 54 | 0.27551 | 52 | 0.001054 | 51 | 157 | 57 |
| 41 | *Alcaligenes* | 1 | 55 | 0 | 55 | 0 | 55 | 165 | 58 |

**Supplemental Table 2.2.** The ranks and values of each genus in the SNI group based on betweenness centrality, closeness centrality, and degree centrality.

| OTU | Genus | Degree centrality score | Rank based  on  degree centrality  score | Closeness centrality  score | Rank  based  on  closeness centrality  score | Betweeness centrality score | Rank  based  on  betweeness centrality score | Total Rank | Final rank |
| --- | --- | --- | --- | --- | --- | --- | --- | --- | --- |
| 53 | *Staphylococcus* | 30 | 2 | 0.574713 | 3 | 0.115747 | 1 | 6 | 1 |
| 7 | *Ruminococcaceae_uncultured* | 32 | 1 | 0.543478 | 7 | 0.10805 | 2 | 10 | 2 |
| 58 | *Turicibacter* | 25 | 5 | 0.549451 | 6 | 0.082056 | 3 | 14 | 3 |
| 33 | *Ruminococcus* | 26 | 4 | 0.609756 | 2 | 0.045515 | 10 | 16 | 4 |
| 30 | *Mucispirillum* | 22 | 7 | 0.555556 | 5 | 0.059444 | 5 | 17 | 5 |
| 4 | *Bacteroidales_unclassified* | 30 | 3 | 0.666667 | 1 | 0.025343 | 22 | 26 | 6 |
| 9 | *Firmicutes_unclassified* | 19 | 11 | 0.505051 | 11 | 0.039362 | 11 | 33 | 7 |
| 5 | *Ruminococcaceae_unclassified* | 21 | 8 | 0.462963 | 21 | 0.054642 | 7 | 36 | 8 |
| 23 | *Barnesiella* | 16 | 19 | 0.510204 | 10 | 0.05149 | 9 | 38 | 9 |
| 1 | *Lachnospiraceae_unclassified* | 23 | 6 | 0.568182 | 4 | 0.014449 | 30 | 40 | 10 |
| 29 | *Parabacteroides* | 18 | 14 | 0.485437 | 14 | 0.033292 | 12 | 40 | 11 |
| 20 | *Oscillibacter* | 20 | 9 | 0.434783 | 29 | 0.054735 | 6 | 44 | 12 |
| 22 | *Bacteroides* | 15 | 20 | 0.49505 | 12 | 0.029306 | 15 | 47 | 13 |
| 51 | *Clostridium_unclassified* | 20 | 10 | 0.485437 | 15 | 0.023442 | 24 | 49 | 14 |
| 34 | *Candidate_division_TM7* | 15 | 23 | 0.471698 | 20 | 0.053482 | 8 | 51 | 15 |
| 11 | *Lachnospiraceae_uncultured* | 18 | 12 | 0.471698 | 19 | 0.026233 | 21 | 52 | 16 |
| 49 | *Streptococcus* | 15 | 24 | 0.454545 | 24 | 0.067679 | 4 | 52 | 17 |
| 14 | *Bacteroidetes_unclassified* | 18 | 16 | 0.515464 | 9 | 0.021133 | 28 | 53 | 18 |
| 31 | *Hydrogenoanaerobacterium* | 18 | 13 | 0.458716 | 22 | 0.030498 | 18 | 53 | 19 |
| 10 | *Ruminococcaceae_Incertae_Sedis* | 18 | 15 | 0.490196 | 13 | 0.01472 | 26 | 54 | 20 |
| 2 | *Bacteroidales_S24-7* | 17 | 17 | 0.537634 | 8 | 0.011255 | 33 | 58 | 21 |
| 54 | *Desulfovibrio* | 13 | 31 | 0.485437 | 16 | 0.030957 | 14 | 61 | 22 |
| 32 | *Roseburia* | 15 | 21 | 0.431034 | 31 | 0.032031 | 13 | 65 | 23 |
| 48 | *Bifidobacterium* | 15 | 25 | 0.485437 | 18 | 0.023676 | 23 | 66 | 24 |
| 45 | *Erysipelotrichaceae_Incertae_Sedis* | 16 | 18 | 0.45045 | 25 | 0.02297 | 25 | 68 | 25 |
| 26 | *Oscillospira* | 15 | 22 | 0.446429 | 27 | 0.026324 | 20 | 69 | 26 |
| 21 | *Acetanaerobacterium* | 14 | 29 | 0.485437 | 17 | 0.019491 | 27 | 73 | 27 |
| 15 | *Erysipelotrichaceae_unclassified* | 13 | 30 | 0.446429 | 28 | 0.031567 | 17 | 75 | 28 |
| 38 | *Parasutterella* | 14 | 28 | 0.458716 | 23 | 0.014173 | 32 | 83 | 29 |
| 25 | *Allobaculum* | 12 | 34 | 0.396825 | 39 | 0.029213 | 16 | 89 | 30 |
| 3 | *Bacteria_unclassified* | 14 | 26 | 0.42735 | 34 | 0.005155 | 38 | 98 | 31 |
| 19 | *Clostridiales_Family_XIII_Incertae_Sedis* | 12 | 32 | 0.42735 | 35 | 0.011934 | 31 | 98 | 32 |
| 28 | *Akkermansia* | 12 | 33 | 0.420168 | 36 | 0.014698 | 29 | 98 | 33 |
| 55 | *Mollicutes_unclassified* | 10 | 37 | 0.446429 | 26 | 0.008011 | 35 | 98 | 34 |
| 17 | *Lactobacillus* | 11 | 35 | 0.431034 | 32 | 0.005056 | 40 | 107 | 35 |
| 56 | *Actinobacteria_unclassified* | 8 | 46 | 0.35461 | 44 | 0.027212 | 19 | 109 | 36 |
| 27 | *Clostridium_Candidatus_Arthromitus* | 9 | 38 | 0.434783 | 30 | 0.004413 | 44 | 112 | 37 |
| 6 | *Clostridiales_unclassified* | 14 | 27 | 0.316456 | 48 | 0.00444 | 43 | 118 | 38 |
| 35 | *Lachnospira* | 8 | 45 | 0.393701 | 40 | 0.010638 | 34 | 119 | 39 |
| 12 | *Mollicutes_RF9* | 9 | 40 | 0.37037 | 41 | 0.005965 | 39 | 120 | 40 |
| 18 | *Moryella* | 9 | 41 | 0.406504 | 37 | 0.004616 | 42 | 120 | 41 |
| 57 | *Eubacterium* | 9 | 39 | 0.396825 | 38 | 0.004203 | 45 | 122 | 42 |
| 59 | *Peptococcaceae_unclassified* | 10 | 36 | 0.359712 | 42 | 0.003707 | 46 | 124 | 43 |
| 24 | *Coprobacillus* | 8 | 44 | 0.328947 | 46 | 0.006675 | 36 | 126 | 44 |
| 16 | *Coriobacteriaceae_unclassified* | 8 | 43 | 0.357143 | 43 | 0.004853 | 41 | 127 | 45 |
| 50 | *Clostridia_unclassified* | 7 | 47 | 0.431034 | 33 | 0.001446 | 50 | 130 | 46 |
| 52 | *Escherichia* | 6 | 48 | 0.324675 | 47 | 0.006617 | 37 | 132 | 47 |
| 13 | *Adlercreutzia* | 8 | 42 | 0.263158 | 50 | 0.001897 | 48 | 140 | 48 |
| 44 | *Acinetobacter* | 4 | 50 | 0.342466 | 45 | 0.001468 | 49 | 144 | 49 |
| 8 | *Lachnospiraceae_Incertae_Sedis* | 5 | 49 | 0.314465 | 49 | 0.002069 | 47 | 145 | 50 |
| 40 | *Porphyromonadaceae_unclassified* | 2 | 51 | 0 | 51 | 0 | 51 | 153 | 51 |
